# Metal–Organic Frameworks: Unlocking New Frontiers in Cardiovascular Diagnosis and Therapy

**DOI:** 10.1002/advs.202416302

**Published:** 2025-04-24

**Authors:** Qilu Wu, Yuxiao Feng, Mathilde Lepoitevin, Meng Yu, Christian Serre, Jun Ge, Yuan Huang

**Affiliations:** ^1^ Key Lab for Industrial Biocatalysis Ministry of Education Department of Chemical Engineering Tsinghua University Beijing 100084 P. R. China; ^2^ Institut des Matériaux Poreux de Paris ENS ESPCI Paris CNRS PSL University Paris 75005 France; ^3^ NMPA Key Laboratory for Research and Evaluation of Drug Metabolism & Guangdong Provincial Key Laboratory of New Drug Screening & Guangdong‐Hongkong‐Macao Joint Laboratory for New Drug Screening School of Pharmaceutical Sciences Southern Medical University Guangzhou 510515 P. R. China; ^4^ State Key Laboratory of Green Biomanufacturing Beijing 100084 P. R. China; ^5^ Cardiac Surgery Centre Fuwai Hospital National Center for Cardiovascular Diseases Chinese Academy of Medical Sciences Peking Union Medical College No.167 North Lishi Road, Xicheng District Beijing 100037 P. R. China

**Keywords:** biocompatibility, cardiovascular disease, diagnosis, metal–organic frameworks, therapy

## Abstract

Cardiovascular disease (CVD) is one of the most critical diseases which is the predominant cause of death in the world. Early screening and diagnosis of the disease and effective treatment after diagnosis play an important role in the patient's recovery. Metal–organic frameworks (MOFs), a kind of hybrid ordered micro or meso‐porous materials, constructed by metal nodes or clusters with organic ligands, due to their special features like high porosity and specific surface area, open metal sites, or ligand tunability, are widely used in various areas including gas storage, catalysis, sensors, biomedicine. Recently, advances in MOFs are bringing new developments and opportunities for the healthcare industry including the theranostic of CVD. In this review, the applications of MOFs are illustrated in the diagnosis and therapy of CVD, including biomarker detection, imaging, drug delivery systems, therapeutic gas delivery platforms, and nanomedicine. Also, the toxicity and biocompatibility of MOFs are discussed. By providing a comprehensive summary of the role played by MOFs in the diagnosis and treatment of CVDs, it is hoped to promote the future applications of MOFs in disease theranostics, especially in CVDs.

## Graphical abstract

Metal–organic frameworks (MOFs), are composed of metal ions and bridging organic ligands, are recognized as an excellent platform for host‐gust chemistry. Its rich structural composition, modifiable sites, and multilevel pore distribution can provide delivery and release of signaling molecules, probes, and therapeutic drugs, which exhibit great medical value in the diagnosis and treatment of CVDs.

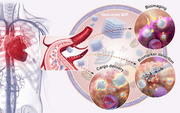

## 1 Introduction

Cardiovascular disease (CVD) is a collective term for a range of heart and blood vessel disorders, which is the predominant causes of death in the world. As of 2020, the number of annual deaths from CVD has risen to 19.05 million globally, a 19% increase from 2010.^[^ ^1^ ^]^ CVD encompasses many types of diseases such as coronary artery disease, stroke, hypertension, heart failure, vascular disease, etc., presenting a complex range of morbid symptoms and causes.^[^ ^1^, ^2^ ^]^ Therefore, the early diagnosis and treatment of CVD are crucial, because they can help patients achieve a longer survival time and better prognosis.^[^ ^3^ ^]^ Along with the escalating public healthcare burden caused by CVD, efforts are being made to find more effective diagnostic and theranostics alternatives to existing technologies.

**Figure 1:**
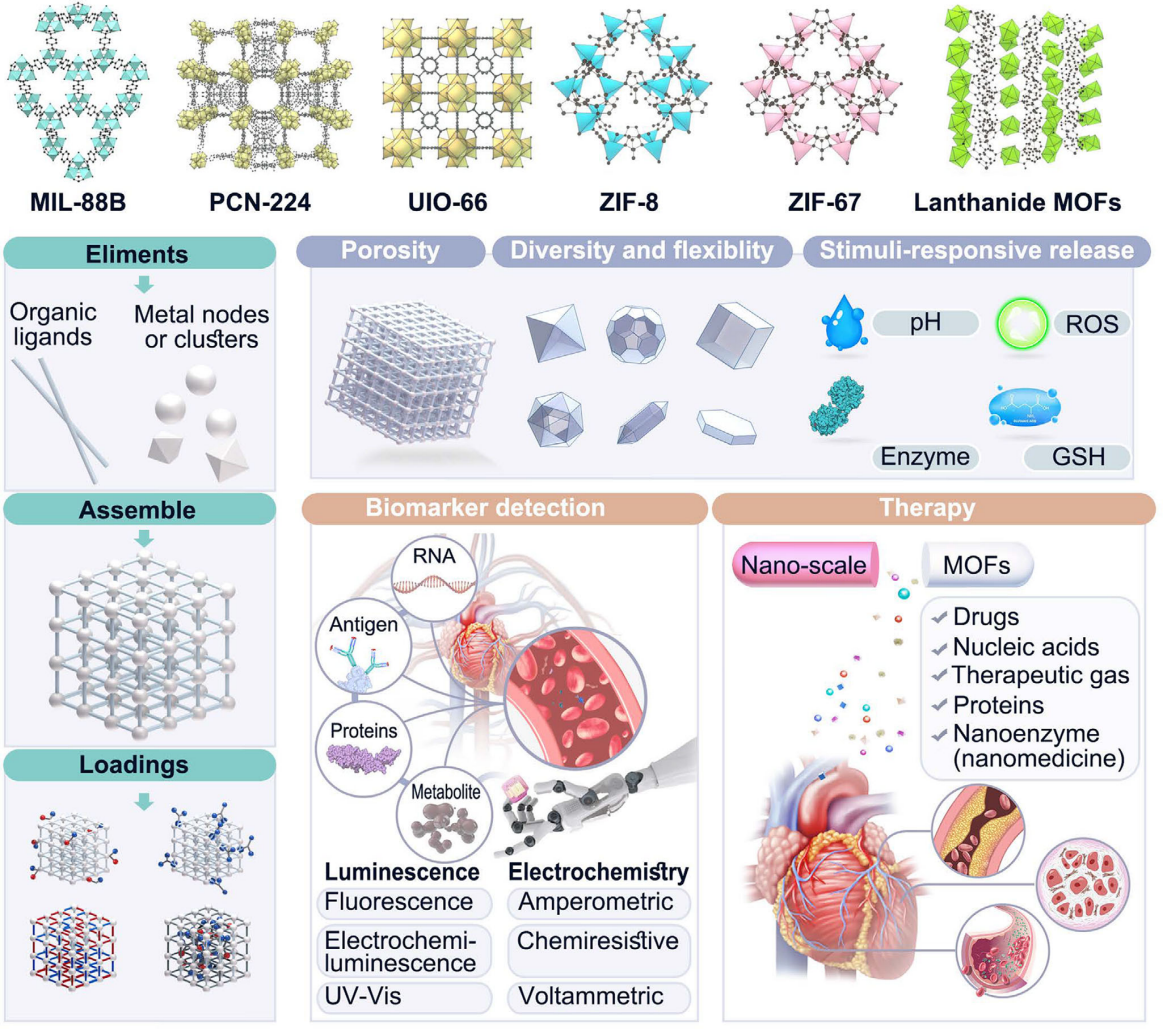
Examples of the structures of MOFs that are commonly used in CVD theranostics and schematic illustration of the synthesis, features, and functions of MOFs for CVD diagnosis and treatment.

Metal–organic frameworks (MOFs) are composed of metal ions and bridging organic ligands. They have been recognized as an excellent platform for host–guest chemistry,^[^ ^4^ ^]^ through their regular and highly tunable pore structure, associated with an enormous variability of i) their inorganic secondary building units, ii) organic ligands and linkers. This results in an unprecedented structural (topology, pore size, shape) and chemical (acid‐base, polar/apolar…) diversity that can further be refined through multiple functionalization approaches (e.g., post‐synthetic modification, multivariates). MOFs are considered for many applications such as gas storage, separation, catalysis, or biomedicine….^[^ ^5^ ^]^ The atomic level localization of the inorganic and organic moieties delimiting their micro or meso‐pores has shown to be of utmost interest to tune the forces (electrostatic, dispersive…) that drive the host–guest interactions at a level never reached before for any type of porous solids.^[^ ^6^ ^]^ Leading to breakthroughs for separations in the gas phase (e.g., N_2_/CH_4_,^[^ ^7^ ^]^ CO_2_/N_2_^[^ ^8^ ^]^…) or more recently for remediation in the liquid phase^[^ ^9^ ^]^ or chiral separation.^[^ ^10^ ^]^ It is exploited in biomedicine for controlled encapsulation and release of challenging drugs or biomolecules.^[^ ^11^ ^]^ Recently, advances in MOFs are bringing new developments and opportunities for the healthcare industry including the theranostics of CVD. The applications of MOFs in CVD can be divided into two main areas: diagnosis^[^ ^12^ ^]^ and therapy.^[^ ^13^ ^]^ In addition, MOFs have also been applied to surface modification of cardiovascular stent materials to achieve better operation effects.^[^ ^14^ ^]^ Furthermore, some MOFs can act as fluorescence nanoprobes for diagnostic imaging.^[^ ^15^ ^]^ The structure of common MOFs used for CVD diagnosis and treatment is shown in **Figure** **1**.

In this review, we will focus on the application of MOFs in CVD and related medical aspects, and the outline of this paper is shown in Figure 1. First, their application in CVDs diagnosis will be introduced prior to discussing their use in the treatment of CVDs. More importantly, advanced research progress and achievements will be elaborated in detail. Some of the major studies of MOFs for CVD diagnosis and treatment are listed in **Table** **1**. We are committed to applying a comprehensive review of MOFs and their application in CVDs, from theoretical foundations to practical applications, providing a reference for the future development of this nanomaterial in the healthcare industry.

**Table 1:** MOFs for CVD diagnosis and treatment.

Application	Type of MOF	Specific MOF	Function	Effect	Disease	Refs.
Diagnosis	Materials of Institute Lavoisier frameworks (MIL)	NH_2_‐MIL‐125(Ti)	Electrochemical Immunosensor	Detection of Galectin‐3 (Gal‐3)	Heart failure	[16a]
		NH_2_‑MIL‐101(Fe)	Electrochemiluminescence Immunosensor	Detection of Cardiac Troponin I (cTnI)	Acute myocardial infarction	[17]
		MIL‐101(Fe)	Electrochemical biosensor	Detection of miR‐721	Myocarditis	[12a]
		MIL‐88B(Fe)	Optical biosensor	Detection of miR‐21, miR‐499 and miR‐133a	Acute myocardial infarction	[18]
		MIL‐101(Fe)	Electrochemical Immunosensor	Detection of Gal‐3	Heart failure	[16b]
	University of Oslo (UiO) MOFs	UiO‐68‐ol	Optical biosensor	Detection of HClO	CVD	[19]
		UiO‐66	Electrochemical Immunosensor	Detection of cTnI	Myocardial injury	[16c]
		UiO‐66	Multiplexed lateral flow assay (LFA)	Detection of Heart‐type fatty acid binding protein (h‐FABP) and cTnT	Acute myocardial infarction	[20]
	PCN	PCN‐224(Zr)	Fluorescence imaging (In vivo)	Evaluation of pH and phosphate levels in blood	Atherosclerosis	[15a]
		PCN‐224(Zr)	Fluorescence imaging (In vivo)	Detection of protein phosphorylation and glucose levels in blood	Atherosclerosis	[15b]
		PCN‐222(Mn)	MRI (In vivo)	‐	Atherosclerosis	[21]
	Lanthanide MOFs	Eu‐MOFs	Optical biosensor	Detection of creatine kinase (CK)	Acute myocardial infarction	[12b]
		Ce‐ MOFs	Electrochemiluminescence Immunosensor	Detection of proprotein convertase subtilisin/Kexin type 9 (PCSK9)	CVD	[22]
		Eu‐MOFs	Optical biosensor	Detection of creatine kinase isoenzyme (CK‐MB), myoglobin (Mb), cTnI and aspirin	Acute myocardial infarction	[23]
		Eu‐MOFs	Optical biosensor	Detection of Trimethylamine‐N‐oxide (TMAO)	CVD	[24]
	Zeolitic imidazolate framework (ZIF)	ZIF‐67	Electrochemiluminescence Immunosensor	Detection of cTnI	Acute myocardial infarction	[25b]
		ZIF‐67	Electrochemical biosensor	Detection of C‐reactive protein (CRP)	CVD	[26]
	Ru‐MOFs	Ru‐MOFs	Electrochemiluminescence Immunosensor	Detection of cTnI	Acute myocardial infarction	[27]
Therapy	MIL	MIL‐47(V)	Nanozyme (In vivo)	ROS scavenging	CVD	[28b]
		MIL‐88B(Fe)	NO Delivery (In vivo)	Vasodilation	Chronic CVD	[29]
	Cu‐MOFs	Cu‐MOFs with polydopamine coating	Coating of cardiovascular stents to reduce thrombosis (In vivo)	NO catalytic generation and Cu^2+^ delivery	Stent implantation	[30a]
		CuSURMOFs	Coating of cardiovascular stents to reduce thrombosis (In vivo)	NO catalytic generation	Stent implantation	[31]
		M199 MOFs	Entrapment of MOFs within PCL materials (In vivo)	NO catalytic generation	Stent implantation	[32]
		Cu‐MOFs	Coating of cardiovascular stents to reduce thrombosis	NO catalytic generation	Stent implantation	[30b]
	UiO	Defective UiO‐66	Delivery of Chloroquine diphosphate (CQ)	Cardiac cells (H9C2)	Arrhythmia	[33]
		UiO‐66	Delivery of rapamycin and IL‐1Ra (In vivo)	Reduced atherosclerosis plaques in coronary arteries, carotid arteries, and aortas	Atherosclerotic CVD	[34a]
		UiO‐66	Delivery of superoxide dismutase (SOD) (In vivo)	ROS scavenging	Acute myocardial infarction	[13]
	ZIF	ZIF‐8	Delivery of losartan potassium (In vivo)	Atherosclerotic target tissue	Atherosclerosis	[35b]
		ZIF‐90	Delivery of Zn^2+^ (In vivo)	Endothelial Cells	Ischemic diseases	[36]
		ZIF‐8	Delivery of antisense oligonucleotides (ASOs) for gene silencing (In vivo)	Transportation of ASOs against microRNA‐155to the endothelial cells in atherosclerotic lesions	Atherosclerosis	[35a]
	Bimetallic MOFs	MOF‐818(Cu, Zr)	Nanozyme	ROS scavenging	Oxidative stress	[37]
		Cu‐TCPP‐Mn	Nanozyme (In vivo)	ROS scavenging	Acute myocardial infarction	[38]
	PCN	PCN‐222(Mn)	Nanozyme and delivery of curcumin (In vivo)	Atherosclerotic target tissue	Atherosclerotic CVD	[21]

## 2 MOFs for Diagnosis of CVD

### 2.1 Biosensor for Biomarker Detection

Currently, the diagnosis of CVDs is mainly based on conventional biomarker testing and imaging methods.^[^ ^39^ ^]^ However, these methods usually exhibit low signal‐to‐noise ratio (SNR), low bioavailability, and nonspecific distribution of drugs, which are still key issues that need to be overwhelmed. In general, early and on‐time diagnosis of CVDs is crucial for the prevention and treatment of disease. Because it can achieve better recovery outcomes and avoid serious physical trauma or even death caused by the progressed condition. Conventional diagnostic techniques for CVD can be divided into two categories: invasive‐based techniques (e.g., coronary angiography) and non‐invasive‐based methods (e.g., computerized tomography, magnetic resonance imaging, electrocardiogram, ultrasound). Although these methods have been widely used and exhibit good reliability, they are still not enough to satisfy the demand for accurate and early disease screening and diagnosis. At the same time, invasive diagnostic methods can cause the risk of complications which is not desirable. Therefore, researchers have been seeking more accurate, fast, and non‐invasive diagnostic methods for CVDs for a long time. Recently, as an emerging and promising nanomaterial, MOFs have shown the potential to play a significant role in the diagnostic fields, especially in the field of biosensing for biomarker detection in the diagnosis of disease.

**Figure 2:**
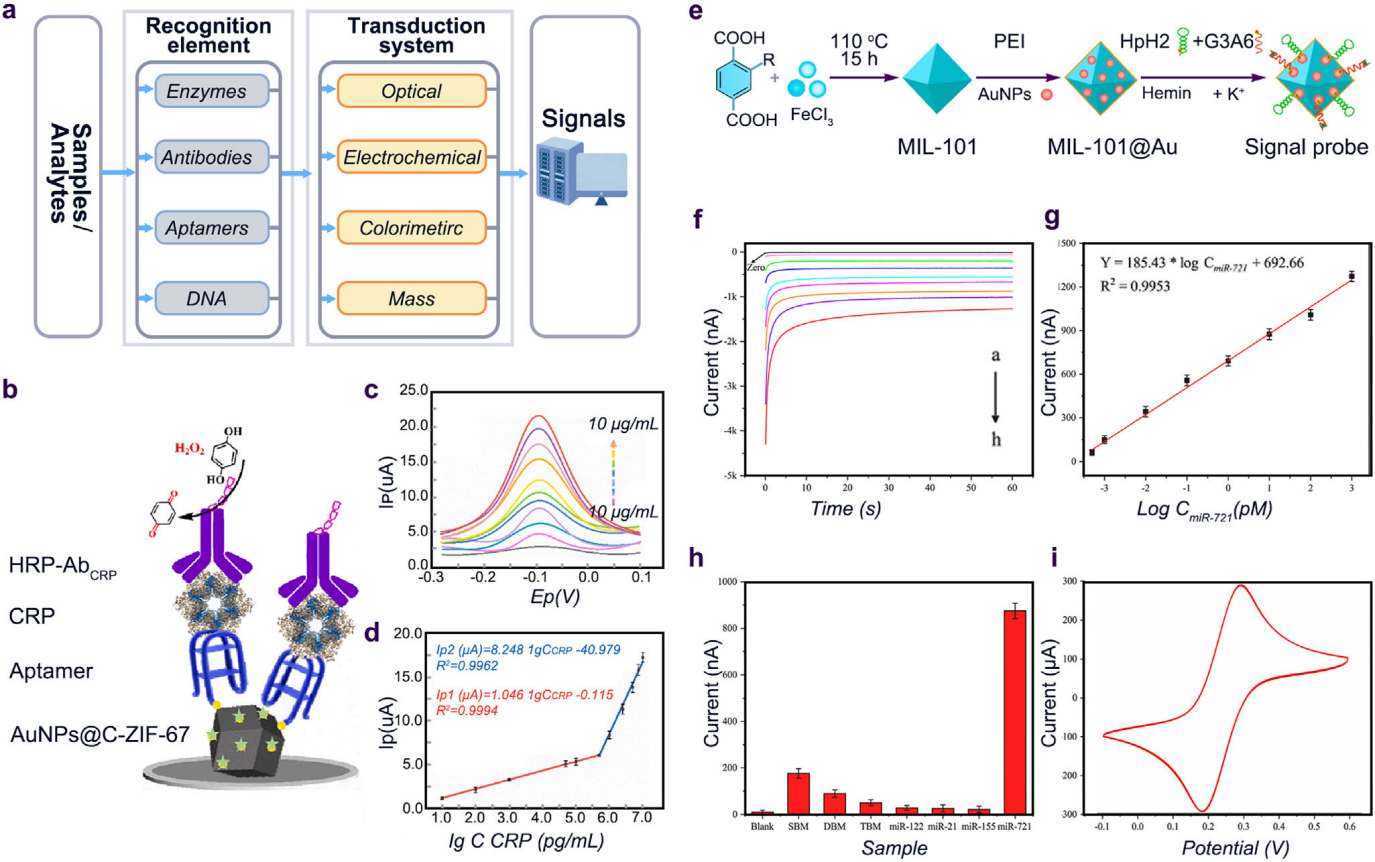
a) Schematic diagram of biosensor. b–d) The strategy of building the aptamer‐antibody sandwich for CRP assay; DPV curves of the proposed sandwich‐type sensor toward CRP with a series of concentrations; Corresponding linear calibration curve between the peak current and the logarithm of concentration. Reproduced with permission.^[^ ^26^ ^]^ Copyright 2022, Elsevier. e–i) Preparation of signal probe (MOF@Au@HpH2‐G3/hemin) of electrochemical biosensors: 0 fm, 0.5 fm, 1 fm, 10 fm, 100 fm, 1 pm, 10 pm, 100 pm, and 1 nm); Calibration curve of different concentrations of miR‐721; Selectivity of the biosensor; Stability of the biosensor. Reproduced with permission.^[^ ^12a^ ^]^ Copyright 2022, Elsevier.

As mentioned earlier, MOFs have been proposed in the field of biomedicine due to their unique properties. With the development of nanotechnology, using molecular probes to detect physiological processes, which is known as biosensing, has become a promising technique for disease diagnosis.^[^ ^15^, ^40^ ^]^ In general, a biosensor consists of four main components: samples/analytes, recognition elements, a transduction system, and a signal output device (**Figure** **2**a). It detects specific components based on the variation of their concentrations, properties, or types caused by the disruption of the physiological environment during illness by specific probes.^[^ ^41^ ^]^ In the past few decades, MOFs based biosensors have made significant progress in the biomedical field by utilizing specific physical or chemical interactions between biomarkers and MOFs to generate detectable signals for detection.^[^ ^42^ ^]^ Throughout the occurrence and progression of CVD, various biomarkers are connected to the physical condition. For example, C‐reaction protein (CRP), an inflammatory marker, is associated with plaque instability and rupture,^[^ ^26^ ^]^ while cardiac troponin (cTn) is essential for diagnosing acute myocardial infarction as it indicates myocardial necrosis.^[^ ^20^ ^]^ However, self‐quenching and leaching are common issues in these small molecular biosensors which can affect accuracy and sensitivity, thus limiting the development of this technique to a certain extent. Benefiting from the flexible combination and multifunctionality of MOFs, MOF‐based biosensors can overcome the above problems and construct a high‐resolution, sensitive, and accurate biosensing system for the diagnosis of CVD.

#### 2.1.1 Electrochemical Biosensor

The electrochemical biosensor is an analytical device that includes the combination of a biological recognition element and an electrochemical transduction system, which provides sensitive and specific detection of analytes through direct contact between the two elements.^[^ ^43^ ^]^ Improving stability, sensitivity, reproducibility, and multiplexing are key points in the development of biosensors. Therefore, a stable detection platform is particularly important for electrochemical sensors, as it affects the stability and sensitivity of the entire detection system. Due to the large surface area of MOFs, many electrochemical transduction elements, such as Au nanoparticles (AuNPs), can be modified on MOFs to enhance their conductivity, promote electron transfer between detection molecules and electrodes, and further improve the sensitivity and detection limit of the system.^[^ ^16^ ^]^ For example, Tang et al. constructed a sandwich‐type immunosensor consisting of the N‐doped graphene nanoribbons immobilized Fe‐based MOFs which were decorated with AuNPs (NGNRs‐Fe‐MOFs@AuNPs) as the substrate platform.^[^ ^16b^ ^]^ Their results indicated that more primary antibodies were captured, and electron transfer in the sensor was significantly accelerated due to the large specific surface area and abundant active sites of Fe‐MOFs. Based on this platform, the immunosensor exhibited a linear response with great sensitivity, stability, reproducibility, and selectivity toward the heart failure biomarker Galectin‐3 (Gal‐3). Meanwhile, a carbonized Co‐based zeolitic imidazolate framework (ZIF‐67) had also been synthesized as a host material to load more AuNPs, which increased the multielectron transfer and mass transport, leading to higher aptamer functionalization and electron transfer for the catalytic reaction of analytes (Figure 2b–d).^[^ ^26^ ^]^

In the process of electrochemical biosensing, the signal is usually weak and susceptible to noise and distortion. Meanwhile, the selectivity and specificity of sensors still mainly rely on the antigen‐antibody reactions, which brings several problems, such as high production costs of antibodies, instability in high temperatures, complicated chemical modifications, and limited detection efficiency.^[^ ^44^ ^]^ Therefore, amplifying electrochemical signals is an important step in the exploitation of electrochemical biosensors. Luo et al. developed a cTnI nanoprobe using Fe_3_O_4_@UiO‐66 magnetic MOFs as a label associating with DNA aptamer.^[^ ^16c^ ^]^ Taking advantage of the large surface area of the microporous UiO‐66, more aptamer, Au@Pt nanoparticles, horseradish peroxidase (HRP), and G‐quadruplex/hemin (GQH) DNAzyme were loaded onto the surface of MOFs to form an electrochemical aptamer sensor with an aptamer‐protein‐nanoprobe sandwich‐type structure, which dramatically amplified the electrochemical signal of cTnI detection.^[^ ^16c^ ^]^ Meanwhile, MOFs‐based nanozyme, a class of simulated enzymes that possess both the unique properties of nanomaterials and catalytic functions of enzymes,^[^ ^45^ ^]^ has shown great potential in the construction of electrochemical biosensing systems in CVD diagnosis. For example, among various types of MOFs, materials of institute Lavoisier frameworks (MIL) are fascinating materials as nanozyme that are capable of decomposing H_2_O_2_ to amplify the signals in the biosensing system. In view of this, Li et al. used the nitro‐functionalized Fe‐based mesoporous MOF (MIL‐101) as a nano‐catalyst and carrier to immobilize G‐triplex/hemin DNAzyme to construct a double‐amplified nanozyme signal probe, which exhibited remarkable catalytic activities, hydrothermal and chemical, and long‐term storage stability (Figure 2e).^[^ ^12a^ ^]^ After the electrochemical signals were amplified by decomposing H_2_O_2_, this probe was proved to be effective for miR‐721 (a diagnostic biomarker of acute myocarditis) detection in real biological samples with high sensitivity, accuracy, and stability (Figure 2f–i).^[^ ^12a^ ^]^ Besides, combining MOFs and covalent organic frameworks (COFs) acts as signal amplification nanocomposites significantly and also enhances the amperage signal in the detection of biomarkers. In the detection of antigen GL‐3 (a β‐galactoside protein biomarker for monitoring heart failure risk and death risk), the microporous Ti‐based MOF (Ti‐MOF, NH_2_‐MIL‐125)@COF (Ti‐MOF@COF) composite materials decorated with anti‐GL‐3‐Ab_2_ (anti‐GL‐3‐Ab_2_/Ti‐MOF@COF) not only promoted the permeability of the protein in the pore but also enhanced the stable antigen‐antibody interaction.^[^ ^16a^ ^]^

#### 2.1.2 Optical Biosensor

Optical biosensor is a common type of biosensor that enables the direct, real‐time, and label‐free detection of biomarkers.^[^ ^46^ ^]^ The optical biosensor as a kind of luminescence detection assay, possesses some unique advantages such as high sensitivity, fast detection speed, high selectivity, and stable signal, making it widely used in the field of chemistry and biochemistry.^[^ ^47^ ^]^ Like an electrochemical biosensor, an optical biosensor also contains a biological recognition element that is in direct contact with an optical transducer element.^[^ ^46^ ^]^ Among them, the fluorescent biosensor has started to be applied in the diagnosis of CVDs in the last few years. Lanthanide metal–organic frameworks (Ln‐MOFs) as an attractive type of MOFs,^[^ ^48^ ^]^ can combine the inherent photoluminescence (PL) properties of lanthanides with the unique features of MOFs to act as promising nanoprobes in biosensors.^[^ ^12^, ^49^ ^]^ However, lanthanide ions exhibit a low light absorption rate which means weak luminescence, while to obtain a high signal‐to‐noise ratio biosensing process, it requires sharp PL emissions.^[^ ^50^ ^]^ At the same time, it is difficult to control the structure and size of Ln‐MOF in nanoscale.^[^ ^51^ ^]^ Therefore, the design and synthesis of ideal Ln‐MOFs for biosensing are still facing many challenges.

**Figure 3:**
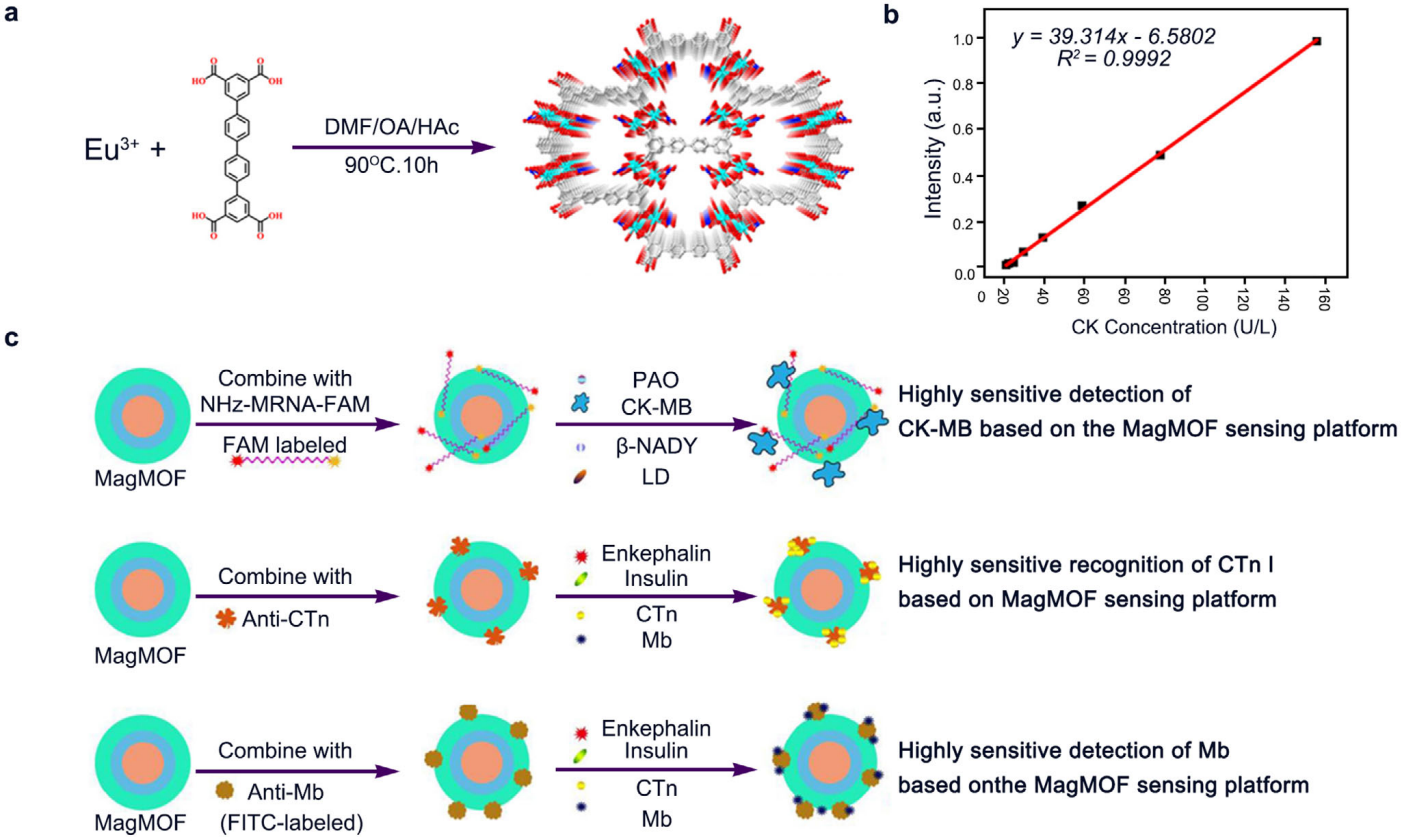
a,b) Synthesis process of Eu–QPTCA: the nanoparticles were produced by the reaction between Eu(CH_3_COO)_3_ and H_4_QPTCA in a mixed solution under 90 °C for 10 h; Relationship between the emission intensity of Eu‐QPTCA nanoparticles and the concentration of CK for CK detection. Reproduced with permission.^[^ ^12b^ ^]^ Copyright 2019, American Chemical Society. c) Fe_2_O_3_@SiO_2_@Eu‐MOF@Tb‐MOF nanoballs as a sensing platform for CK‐MB, CTn I, and Mb biomarkers detection. Reproduced with permission.^[^ ^23^ ^]^ Copyright 2022, American Chemical Society.

Acute myocardial infarction, the leading cause of death in CVD, is myocardial necrosis caused by acute persistent ischemia and hypoxia in the coronary artery.^[^ ^52^ ^]^ Biomarker detection is also the common method for the diagnosis of this disease. In order to obtain more intuitive and accurate detection results, the detection of relevant biomarkers through fluorescent probes has become an intuitionistic diagnostic technique. Creatine kinase (CK) is an important kinase related to intracellular energy function. The level of CK in normal human serum is relatively low, but after coronary occlusion, numerous CK will be released into the bloodstream, resulting in a significant increase in serum CK levels in a short period of time.^[^ ^53^ ^]^ Therefore, CK can serve as an important biomarker for indicating acute myocardial infarction. At present, the reference method for the determination of CK activity is based on UV–vis absorption spectroscopy,^[^ ^54^ ^]^ while the sensitivity and specificity are still unsatisfactory. Fortunately, the organized building blocks and numerous functional groups of MOFs can regulate the intensity and position of fluorescence. In addition, the high specific surface area is also conducive to the attraction of the surface to the target substance, which is advantageous to the construction of a sensing system.^[^ ^55^ ^]^ Thus, as shown in **Figure** **3**a, Li and co‐workers synthesized a nanoscale Ln‐MOF nanoprobe based on time‐resolved luminescent lanthanide MOF (Eu‐QPTCA) through a simple self‐assembly process, which can produce strong red emission under UV excitation. Then the PL characteristics of the MOFs were applied to detect the existence of CK, and the results in Figure 3b showed that the concentration of CK had a stable linear relationship with fluorescence intensity, which indicated that the nanoprobe has good stability, selectivity, and reliability in detecting CK activity in human serum.^[^ ^12b^ ^]^

Meanwhile, the self‐assembly capability of MOFs allows nanoparticles to form customizable multi‐shell nanostructures through a layer‐by‐layer construction strategy that can further exert the capabilities of fluorescent chromophores. At the same time, multi‐shell nanoparticles with magnetic cores are easier to prepare and can be quickly separated by magnetic force. The synthesis strategy makes the size and shape of the nanoparticles controllable, which is convenient for adjusting the optical properties of the particles. For instance, Shi et al. synthesized a stratified magnetic core/multi‐shell Fe_2_O_3_@SiO_2_@Eu‐MOF@Tb‐MOF nanoballs.^[^ ^23^ ^]^ Combined with different antibodies or mRNA sequences, these nanoballs can sensitively detect different acute myocardial infarction biomarkers, such as CK, myoglobin (MB), and troponin I (cTnI) (Figure 3c). In addition, the previously mentioned Ln‐MOFs are also capable of detecting other CVD biomarkers like trimethylamine‐N‐oxide,^[^ ^24^ ^]^ hydrogen sulfide, and ascorbic acid.^[^ ^49c^ ^]^ As a result, Ln‐MOFs can act as an excellent nanoprobe in fluorescent biosensors for the detection of CVD biomarkers with outstanding stability, sensitivity, speed, and convenience.

**Figure 4:**
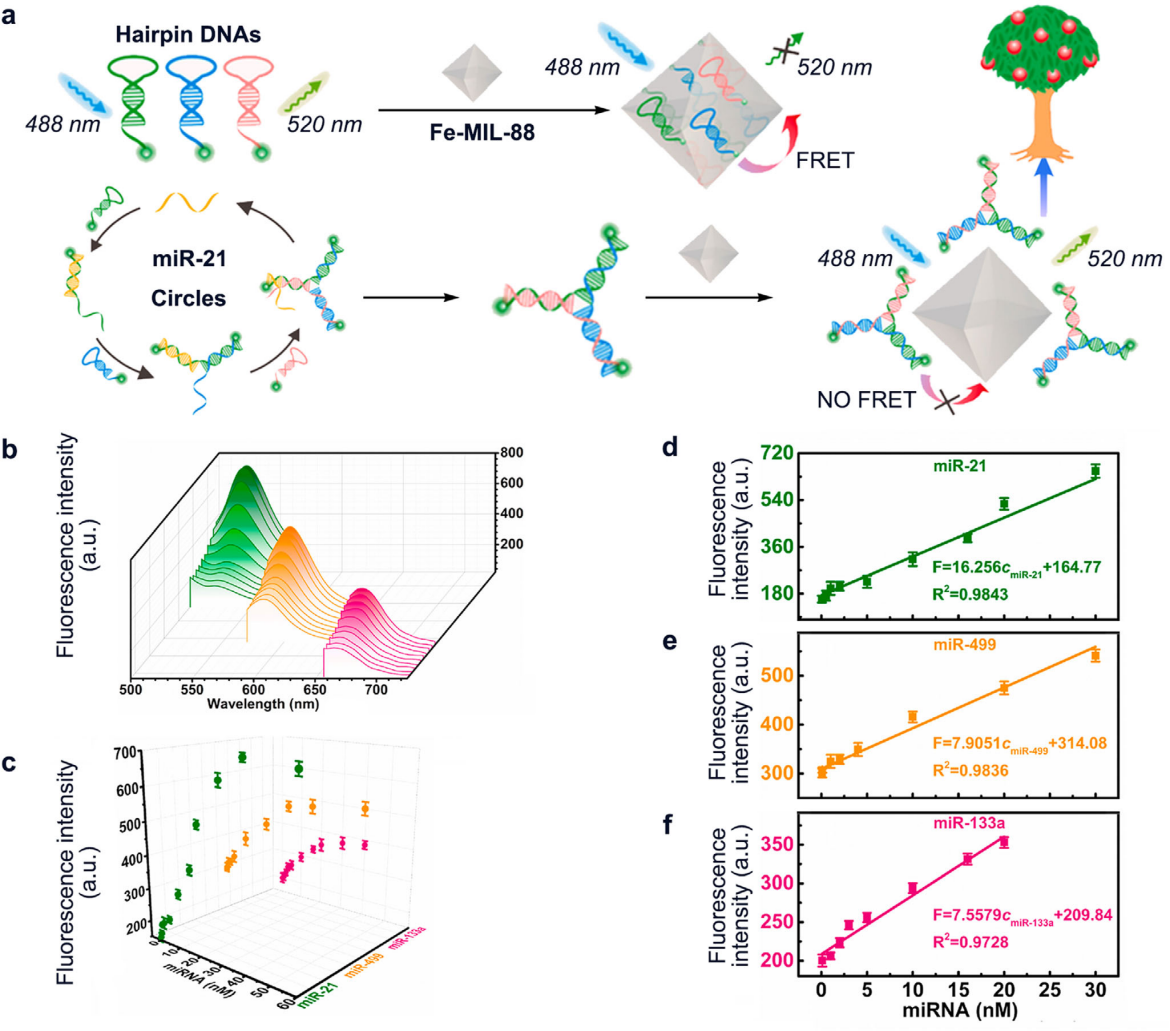
a) The fluorescence of the designed hairpin DNA with the modification terminal end can be quenched by Fe‐MIL‐88, while the target miRNAs appeared, they triggered a catalytic hairpin assembly (CHA) reaction with the corresponding DNA molecular beacons to produce “Y”‐shaped nanostructures. Due to the weak affinity between Fe‐MIL‐88B and the “Y”‐shaped nanostructures, the “Y”‐shaped nanostructures could not be adsorbed on the surface of Fe‐MIL‐88B to quench the fluorescence. b) Fluorescence spectra of FAM/TAMRA/Cy5 with different concentrations of miR‐21 (0.05–60 nm), miR‐499 (0.08–60 nm), and miR‐133a (0.1–40 nm). c) Relationship between the fluorescent intensities and the concentrations of different miRNA. d–f) Calibration curves of different miRNA based on multiple miRNA assay. The fluorescence excitation wavelengths were 488, 552, and 635 nm, respectively. Reproduced with permission.^[^ ^18^ ^]^ Copyright 2022, Elsevier.

Besides, not only Ln‐MOFs exhibit excellent properties in optical biosensors due to the optical properties of the metal nodes, but other types of MOFs can also be applied as powerful tools for the detection of CVD biomarkers. Specifically, through the interaction of van der Waals forces and *π*−*π* stacking, MOFs have a unique adsorption capacity for single‐stranded DNA (ssDNA) than other types of nucleic acid, so these MOFs are able to recognize varieties of dye‐labeled ssDNA by the fluorescence bursting.^[^ ^18^ ^]^ For example, Cheng et al. constructed a biosensor FAM/TAMRA/Cy5 which decorated fluorescent dyes with several hairpin DNAs on their sticky ends, and when hairpin DNAs were adsorbed on the microporous flexible MOF denoted Fe‐MIL‐88B, the fluorescence was quenched by the fluorescence resonance energy transfer effect (FRET). But when target miRNAs were in the presence, they would form a “Y” shape with the hairpin DNA, and their affinity with the Fe‐MIL‐88 was much weaker than the hairpin DNAs only, so the fluorescence would be retained (**Figure** **4**a).^[^ ^18^ ^]^ Their results have shown that within a certain concentration range, the concentrations of different miRNAs and the fluorescence intensities showed an excellent linear relationship (Figure 4b–f). This multiple detection platform is superior to the single detection platform and is beneficial for analyzing miRNAs in complex samples.^[^ ^18^ ^]^ In addition, the modification of the organic ligands of MOFs to obtain redox‐switchable MOFs is also an effective way to synthesize fluorescent probes for the detection of CVD biomarkers. Li et al. constructed a new redox‐switchable microporous Zr MOF called UiO‐68‐ol with a functionalized *p*‐methyloxyphenol‐containing bicarboxylic acid ligand (H_2_L‐ol), which can detect hypochlorous acid (an important reactive oxygen species that is related to the CVD) in living cells.^[^ ^19^ ^]^ Along with the redox process of UiO‐68‐ol, the fluorescence intensity also changed dramatically. After treatment with HClO, the strong fluorescence of H_2_L‐ol was almost quenched. But after reduction, the blue emission was basically restored. The stability and low toxicity of UiO‐type Zr carboxylate MOFs in aqueous media effectively improve the stability and expand the application scenarios of the fluorescent probe, and the large internal pores also promote the contact between the probe and the analyte of interest.^[^ ^19^ ^]^

#### 2.1.3 Electrochemiluminescence Immunosensor

Another important biosensor worth mentioning separately is the electrochemiluminescence (ECL) immunosensor, which is a class of electrochemical biosensors. It is an attractive method for the detection of biomarkers through ECL due to its excellent characteristics such as fast response, low cost, simple operation, high sensitivity, and near‐zero background.^[^ ^56^ ^]^ However, the conventional ECL sensing system suffers from limited sensitivity and the self‐decomposition like luminol‐H_2_O_2_ sensing system, which hinders its development in ultrasensitive and quantitative analysis.^[^ ^25^ ^]^ It is already clear that MOFs are formed by the combinations of metal nodes or clusters with complexing polytopic organic ligands which leads to a porous ordered crystal structure, so molecules can be decorated on the surface of MOFs through various interactions with either the metal nodes or the terminating ligand such as electrostatic adsorption, coordination, and *π*–*π* stacking.^[^ ^57^ ^]^ Therefore, in order to enhance the performance and stability of the ECL immunosensor, this latter crucial emitter can be attached to the surface of MOF nanoparticles to form a more stable structure. Fortunately, it has been found that MOFs can effectively enhance the performance of ECL immunosensor by serving as carriers for ECL emitters to avoid their loss in the liquid phase and improve electrochemical reactions.^[^ ^56^, ^58^ ^]^

Currently, the diagnosis of myocardial infarction mainly relies on the detection of biomarkers, especially cTn isoforms I and T (cTnI and cTnT).^[^ ^40a^ ^]^ Wang et al. synthesized a luminol‐AgNPs@ZIF‐67 nanocomposite which was microporous, although toxic, Co‐based zeolitic imidazolate frameworks ZIF‐67 decorated with luminal‐capped Ag nanoparticles (luminol‐AgNPs) on the surface. It has been applied in a label‐free ECL immunosensor as an emitter with good stability and a great detection limit for cTnI ultrasensitive and early detection.^[^ ^25b^ ^]^ The results demonstrated that ZIF‐67 significantly enhanced the performance of ECL. This improvement was attributed to the porous crystal structure and the atomically dispersed Co^2+^, both of which were conducive to the generation of oxygen radicals. Meanwhile, the high specific surface area of ZIF‐67 made it a great platform to carry AgNPs, avoiding the agglomeration of AgNPs.^[^ ^25b^ ^]^ In addition, a new type of luminophore called 2D semiconductor carbon nitride nanosheets (CNNS) have also been loaded onto the surface of the amino‐functional mesoporous MOF denoted NH_2_‐MIL‐101(Fe) to synthesize a highly efficient ECL luminescent nanomaterial for cTnI detection.^[^ ^17^ ^]^ As shown in Figure 3f, due to the high porosity and the great specificity, NH_2_‐MIL(Fe) could accelerate electron transfer, enhance the loading capacity of CNNS, and serve as a co‐reaction accelerator to improve the decomposition of S_2_O_8_^2−^ which was beneficial for generating a large amount of ECL intermediate SO_4_^•−^ during the biosensing process.^[^ ^17^ ^]^

Moreover, ECL luminophore can be bound to MOFs in the form of ligands, thus enhancing the performance of ECL immunosensor. As a common ECL emitter, tris(2,2′‐bipyridyl)‐ruthenium(II) (Ru(bpy)_3_^2+^) and its derivatives also suffer from issues like limited ECL efficiency and leakage problems.^[^ ^59^ ^]^ So, Yan et al. synthesized 2D functionalized MOF nanosheets with a Ru(bpy)_3_^2+^ derivative (RuMOFNS) called tris(4,4′‐dicarboxylic acid‐2,2′‐bipyridyl)‐ruthenium(II) dichloride (Ru(dcbpy)_3_^2+^) as the organic ligand by a one‐pot method in the aqueous phase.^[^ ^27^ ^]^ This porous RuMOFNS had stronger specificity and could effectively reduce the inactive emission of emitter molecules,^[^ ^60^ ^]^ increase the load of the Ru(dcbpy)_3_^2+^, and prevent leakage of the ECL emitters. Moreover, the 2D structure of RuMOFNS also enhanced the performance of ECL by exposing more accessible active sites on the surface than the pure MOFs crystals, which facilitated the contact with substrate and improved ECL efficiency. A stable ECL immunosensor for detecting cTnI was constructed based on the RuMOFNS, which exhibited excellent properties like high sensitivity, low detection limit, and wide range.^[^ ^27^ ^]^ Besides, organic ligands like 5,10,15,20‐tetrakis (4‐carboxyphenyl) porphyrin (TCPP) can also be introduced into MOFs which showed high ECL and PL efficiency for the detection of cTnI.^[^ ^58b^ ^]^

### 2.2 Imaging

**Figure 5:**
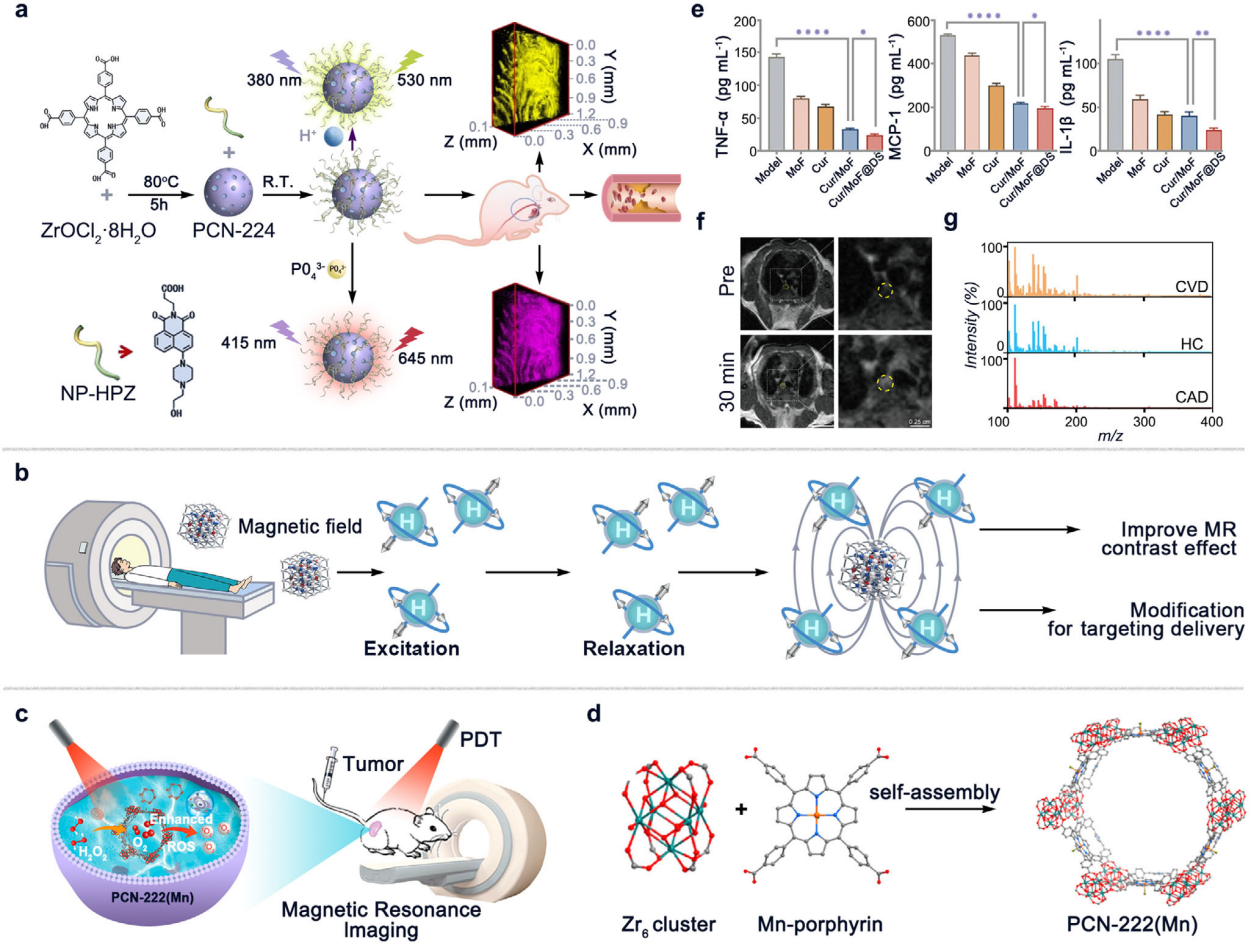
a) The construction of PCN‐NP‐HPZ nanosensor and two‐photon fluorescence imaging of pH level in the aortic wall and phosphorylation level in an aortic inner wall of mice with early atherosclerosis. Reproduced with permission.^[^ ^15a^ ^]^ Copyright 2023, Wiley‐VCH. b) The mechanism and function of MOFs in MRI. c,d) Illustration of MRI‐guided oxygen self‐supplementing photodynamic therapy based on PCN‐222(Mn); The construction of PCN‐222(Mn) framework by Zr_6_ clusters and Mn‐porphyrin. Reproduced with permission.^[^ ^68^ ^]^ Copyright 2019, American Chemical Society. e,f) Levels of inflammatory factors in serum after different treatments; T1‐weighted MRI of the aorta before and after injection of Cur/MOF@DS. The circles marked with dotted lines were contrast‐enhanced regions of atherosclerosis plaque. Reproduced with permission.^[^ ^21^ ^]^ Copyright 2024, Wiley‐VCH. g) Mass spectra of healthy controls (HC), CVD, and CAD samples obtained using a zinc‐based MOF‐assisted LDI MS. Reproduced with permission.^[^ ^71a^ ^]^ Copyright 2023, American Chemical Society.

Protein phosphorylation is closely related to the development of atherosclerosis, which is a major cause of CVD, so monitoring changes in protein phosphorylation sites in vivo is beneficial for diagnosing and treating related diseases. Traditionally, protein phosphorylation sites are detected by biological mass spectrometry which is hard to achieve in situ analysis of protein phosphorylation sites in vivo.^[^ ^15^, ^61^ ^]^ Taking advantage of the tunability of MOFs, it is possible to post‐modify MOFs to construct specific sensitive fluorescent probes for two‐photon fluorescence imaging in vivo. For instance, Tang's research group combined an organic ligand 5,10,15,20‐tetra (4‐carboxyl) porphyrin with Zr^IV^ to synthesize a type of MOF called PCN‐224.^[^ ^15^ ^]^ Then, they modified post‐synthetically PCN‐224 with different functional molecules (e.g., PCN‐NP‐HPZ whose structure and function are shown in **Figure** **5**a) which can achieve two‐photon fluorescence imaging of protein phosphorylation sites along with pH^[^ ^15a^ ^]^ or glucose levels.^[^ ^15b^ ^]^ Through the advantages of low biological background and large penetration depth of two‐photon fluorescence imaging, these post‐modified MOFs successfully monitored the changes of protein phosphorylation sites in the vascular endothelium in the early stage of atherosclerosis, verified the pathogenesis of atherosclerosis, and provided a suitable fluorescence imaging tool for the diagnosis and monitoring of related diseases.

Moreover, nanoscale MOFs also have the potential to be contrast agents for magnetic resonance images (MRI). From MRI a large amount of information about body function and state by detecting water molecules can be obtained, so local water density, cellular environment, retention time of the inner bound water, and rotational correlation time of the contrast agents can all affect the results.^[^ ^62^ ^]^ In terms of MRI diagnostics, Gd‐agents are commonly used as contrast agents in the clinic and are well detected. However, since Gd‐agents contain Gd3^+^ which is a toxic substance that can cause damage to the human body, such as nephrogenic systemic fibrosis, enzyme inhibition, calcium channel blockade, etc.^[^ ^63^ ^]^ Meanwhile, it has been reported into high concentrations of iron‐based contrast agents can also be toxic to cells.^[^ ^64^ ^]^ The structural diversity of MOFs can enable MOFs to carry and bind different kinds of contrast agents for MRI detection. For example, Mn‐MOFs‐74 was designed to overcome the shortcomings of Gd‐agents by using low‐toxicity Mn as the core and contrast agent of MOFs, exhibiting long rotational correlation time and high relaxivity.^[^ ^65^ ^]^ Besides, the porous nature and large specific surface area of MOFs allow for the simultaneous loading of contrast agents and drugs for integrated diagnosis and treatment.^[^ ^66^ ^]^ The mechanism of MRI is shown in Figure 5b. An ideal contrast agent is based on spin reorientation in paramagnetic, which increases the relaxation rate of the water proton relaxation, thereby enhancing the contrast of the image.^[^ ^67^ ^]^ In general terms, imaging agents can be inserted into the structure (metals and/or organic ligands) at the synthesis stage as well as the post‐synthesis stage through adsorption or post‐modification.^[^ ^67b^ ^]^ He et al. constructed a porous MOF nanozyme called PCN‐222(Mn) which is composed of Mn‐porphyrin ligands and Zr^4+^ ions, and the structure is shown in Figure 5d.^[^ ^68^ ^]^ PCN‐222(Mn) has shown high longitudinal relaxivity for MRI due to the high dispersibility of Mn in the MOF and the high water affinity of the surface (Figure 5c). In the meantime, good catalytic activity of PCN‐222(Mn) for generating oxygen has also been confirmed which could enhance the performance of photodynamic therapy in tumor cells.^[^ ^68^ ^]^ More recently, based on former research, a nanozyme‐like MOF platform has been established for MRI imaging and treatment of atherosclerotic CVD. Specifically, curcumin (Cur) as a traditional medicine was loaded in PCN‐222(Mn) with dextran sulfate (DS) modified on the surface (Cur/MOF@DS). PCN‐222(Mn) not only exhibits high relaxivity but also acts as a carrier to deliver drugs with therapeutic effects into target cells which greatly reduces the level of inflammation in vivo (Figure 5e).^[^ ^21^ ^]^ More importantly, the signal of atherosclerosis plaque in T1‐weighted MRI has been significantly enhanced by using Cur/MOF@DS (Figure 5f).^[^ ^21^ ^]^ It further demonstrates that MOF has a strong potential for CVD applications with diagnostic functions especially for plaque MRI and composition analyses. Meanwhile, more types of MOFs have the potential to be used for diagnostic imaging of CVD in the future because of their good relaxivity and unique structures or characteristics, although most of them have not been tested in CVD related models yet. For example as iron(III) carboxylate MOFs like MIL‐88A not only have paramagnetic iron atoms in their component but also possess porous frame structures filled with metal coordination. Horcajada et al. took advantage of the properties and performance of the non‐toxic MOFs to establish new contrast agent with good relaxivity due to the great coordination between the metal sites and the water molecules, exchanges and diffusion between water molecules^[^ ^69^ ^]^; later on, due to the limited intrinsic paramagnetic properties of iron(III) MOFs, Steunou et al proposed to couple biocompatible iron MOFs, here MIL‐100(Fe), with superparamagnetic USPIO (Ultra Small Particles of Iron Oxide), either by post‐synthetic modification (grafting of citrate coated USPIO) or direct synthesis using mild conditions,^[^ ^70^ ^]^ leading to tunable significantly higher relaxivity values of interest for theranostics.

### 2.3 Other

The metabolomic characterization of CVD has impeded the development of related research. Meanwhile, the existing diagnostic methods of CVD still rely on clinical observation and monitoring, which may lead to misdiagnosis. Thus, by developing the application of metabolic profiling, which is the process of measuring molecular biochemical objects, it can effectively provide accurate diagnosis and molecular subtype analysis of CVD. The strategy of combining MOF with laser desorption/ionization mass spectrometry (LDI‐MS) benefits from the high directionality, microporosity, homogeneous surface, and unique conductivity of the MOF nanoplatforms to generate reliable mass spectrometry data (Figure 5g), which can effectively improve serum metabolic profiling and facilitate large‐scale clinical applications.^[^ ^71^ ^]^

## 3 MOFs for Therapy of CVD

Despite considerable efforts, the efficiency of conventional treatments for CVD including systematic administration and surgery is still limited at present. Notably, with the rapid development of synthetic methods and surface modification strategies, MOFs with advanced properties have been synthesized to meet the growing demand for nanomaterials in biomedical applications. The use of MOFs as delivery vehicles for disease treatment is a promising therapeutic tool in the hope of achieving the desired pharmacological effects with minimal side effects. In this section, we will elaborate on the studies of MOFs for the treatment of CVD and related symptoms.

### 3.1 Drug Delivery

**Figure 6:**
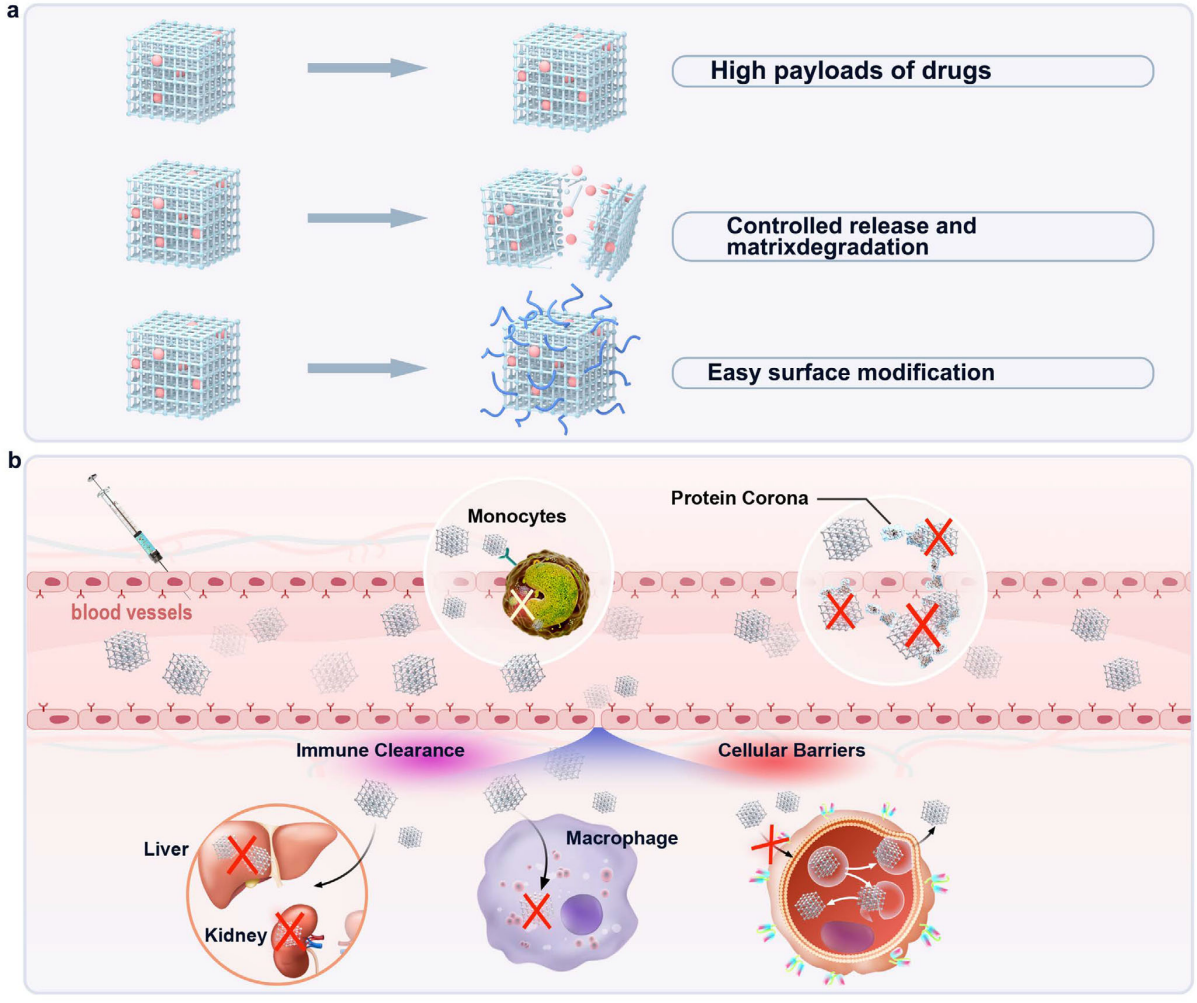
a) Advantages of MOFs as drug‐delivery nanocarriers. b) The biological barriers in MOF‐based drug delivery systems.

Nanotechnology can help overcome the limitations of traditional drug delivery modalities from large‐scale issues (biodistribution) to small‐scale hurdles (intracellular transport) through cell‐specific targeting, molecular transport to specific organelles, and other methods.^[^ ^72^ ^]^ The application of controlled drug delivery systems is a core strategy to improve the efficacy and safety of therapeutic molecules. The main rationale for using a suitable drug delivery system is that it ensures higher and longer drug bioavailability, resulting in improved efficacy. As nanocarriers for therapeutics, MOFs also should meet several requirements that ensure the high efficiency of therapy: 1) high payloads of drugs, 2) controlled release and matrix degradation, 3) easy surface modification (**Figure** **6**a).^[^ ^69^ ^]^ A typical MOFs‐based drug delivery system involves the encapsulation of drug molecules in nano‐cage structures of MOFs to enable therapeutic substance delivery and their sustained release in vivo. May et al. have established a new method to predict the tissue accumulation of nanomedicines by detecting histopathological biomarkers.^[^ ^73^ ^]^ They chose distinct biomarkers like the densities of blood vessels as effective tools that can be related to the concentration of nanomedicines in target tissues by machine learning. After sufficient data feeding to machine learning, the biomarker profile can be stabilized to reflect the accumulation of nanomedicines thus validating the targeting efficiency of the drug in vivo. Nevertheless, regardless of MOF‐based drug delivery systems or other nano‐delivery platforms, most of the current development strategies are focused on improving drug targeting efficiency, and studies have shown that the current targeting efficacy of nanomedicines to the target tissues usually ranges from 0.07 to 7%ID (%ID: per gram of tissue) which seems to be relatively low,^[^ ^74^ ^]^ but this result has not affected the successful promotion of some of the nanomedicines in the market. It is not that the drug delivery systems have not improved drug targeting, while these nanomedicines have other important advantages, such as better tolerability, longer administration time, reduced infusion time, increased drug dosage, and so on. Therefore, developing drug delivery systems that can reach the market and benefit patients needs a vision beyond the limitations of “targeting” and focuses on real‐world therapeutic issues, such as manufacturing protocols, patient selection, and combination therapeutic strategies, to name a few.^[^ ^75^ ^]^

Currently, drug delivery systems are effective in the field of localized therapy, but localized therapy makes it difficult to access organs and tissues such as the heart. Besides, as shown in Figure 6b, there are sequential biological barriers to MOF‐based or similar nanocarrier drug delivery systems. After intravenous administration, nanocarriers may be phagocytosed by the mononuclear phagocyte system (MPS), which is the first obstacle, or lysed and taken up by macrophages, leading to drug aggregation and clearance in the spleen and liver.^[^ ^76^ ^]^ At the same time, serum proteins in the bloodstream can be adsorbed on the surface of nanocarriers to form protein corona, resulting in the inability of the drug to be released properly.^[^ ^77^ ^]^ In addition to this, the cell membrane of the target cell is also a biological barrier. If nanocarriers can enter the target cell successfully, it is still possible that they are unable to complete endosomal escape within the cell, or the drugs are expelled from the cell by the drug‐resistant drug efflux pump.^[^ ^78^ ^]^

**Figure 7:**
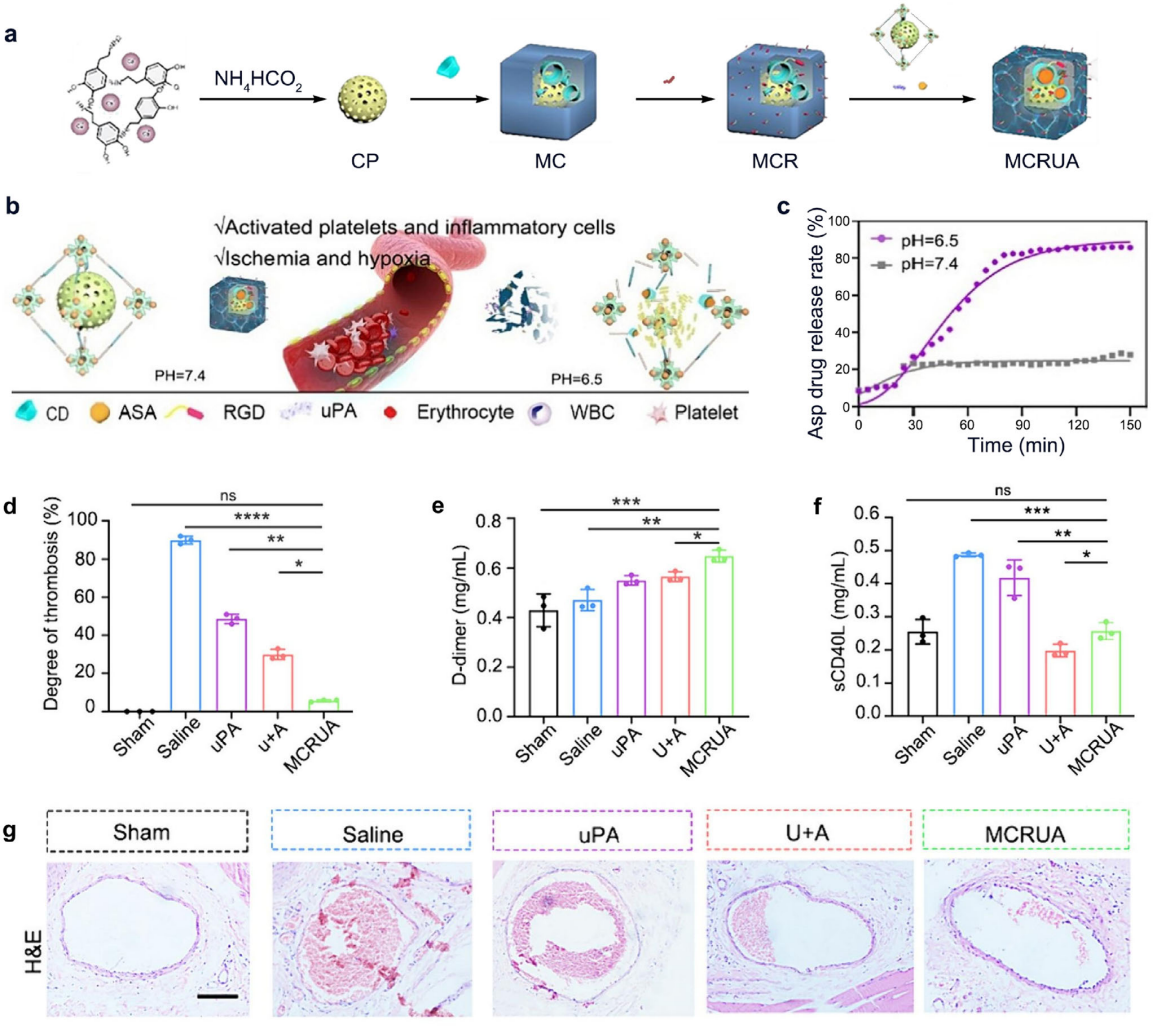
a) Design and synthesis of MCRUA. b) Schematic illustration of pH‐response capability of MCRUA. c) ASA Released by MCRUA. d) Quantification of the thrombus area in each group, *n* = 3. e) The D‐dimer level of mice after different treatments, *n* = 3. f) The sCD40L level of mice after different treatments, *n* = 3. g) Representative histological analysis of the femoral vein after treatment. Scale bar: 200 µm. Reproduced with permission.^[^ ^80b^ ^]^ Copyright 2024, Elsevier.

As a promising nanocarrier, MOFs can adsorb drugs on their surface or encapsulate them inside their pores. Especially the encapsulation of drugs can effectively protect the structure and properties of the drugs from the interference of the external environment, which will ensure their effectiveness before being released. At the same time, the presence of MOFs can also promote slow release and prolong the release time of the drug to achieve a long‐lasting effect. For example, adenosine is an effective small molecule that possesses great anti‐inflammatory ability, while its therapeutic effect always localizes to the releasing point because of the rapid metabolism.^[^ ^79^ ^]^ So, El‐Mehalmey et al. chose several types of microporous MOFs to encapsulate adenosine and achieve the extended release of adenosine in endothelial cells (ECs).^[^ ^79b^ ^]^ Zr‐MOFs (UiO‐66 and UiO‐66‐NH_2_) have exhibited great stability and low cytotoxicity, and the releasing time of adenosine has been extended to nine days to reduce the undesirable side effects because microporous UiO Zr carboxylate MOFs have excellent stability in water but proper (rapid) degradability in buffer system with similarities to the human body's internal environment, which enhances the solubility and bioavailability of the nanocomposite.^[^ ^34^ ^]^ This leads to another important property of MOFs, which is the stimuli‐responsive release in specific environments. A typical example of this is the decomposition of ZIF‐8 in low‐pH environments. ZIF are Zn imidazolates that have a specific pH responsive behavior with good stability at neutral pH but rapid degradation under acidic conditions, sometimes leading to unwanted toxicity side effects due to a burst release of Zn^2+^ ions. Utilizing this feature of ZIF‐8, drug‐loaded ZIF‐8 can transport to the acidic target sites and release the drug responsively in the presence of low pH.^[^ ^35^ ^]^ For example, ZIF‐8 nanoparticles loaded with losartan potassium aggregated and disintegrated under low pH conditions in atherosclerosis tissues to activate autophagy, thereby regulating lipid metabolism and restoring cholesterol homeostasis for anti‐atherosclerosis.^[^ ^35b^ ^]^ Also, in addition to ZIF‐8, there are other MOFs capable of responsive release in vivo.^[^ ^80^ ^]^ Yuan et al. synthesized cyclodextrin MOF nanocarriers (MCRUA) which can sense pH changes in the local ischemic‐inflammatory environment triggering disassembly of the carriers and releasing drugs to dissolve blood clots and reducing inflammation caused by venous thromboembolism (VTE) (**Figure** **7**a–c).^[^ ^80b^ ^]^ The results proved that this pH‐responsive release delivery nanoplatform has an excellent thrombolytic effect on thrombosis (Figure 7d–g).^[^ ^80b^ ^]^ Besides, the selection of drugs to be incorporated in MOFs is usually limited by the pore size distribution and their microporosity, as most mesoporous biocompatible MOFs are not stable enough in aqueous conditions leading to a very fast degradation in body fluids, thus utilizing the special structure of nanocrystalline defective MOFs further expands the application of MOFs in drug delivery.^[^ ^11^, ^33^ ^]^ Well‐developed defective MOFs not only protect the cargo from the invasion of the environment but also enhance molecules or drug loading and the accessibility of the drugs to a certain extent.^[^ ^81^ ^]^

**Figure 8:**
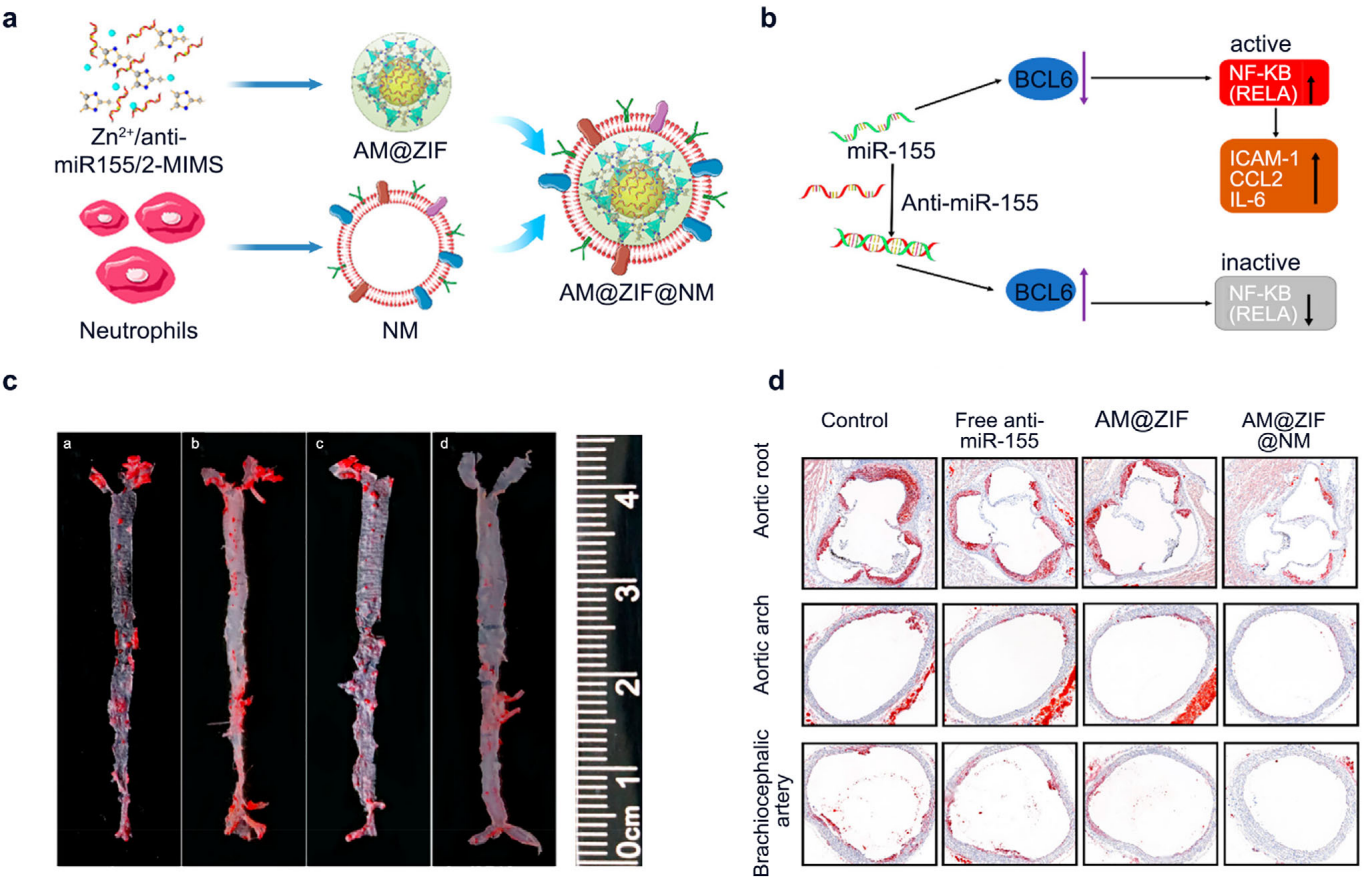
a) Construction of AM@ZIF@NM: containing anti‐miR‐155‐loaded ZIF‐8 (AM@ZIF) core and neutrophil membrane (NM) coating; Illustration of targeted treatment of atherosclerosis with AM@ZIF@NM. Reproduced with permission. b) Schematic diagram of the effect of miR‐155 on NF‐κB signaling pathway. c) Therapeutic efficacy of AM@ZIF@NM NPs in ApoE^–/–^ mice. (a, PBS control; b, Free anti‐miR‐155; c, AM@ZIF; d, AM@ZIF@NM). d) Images of ORO‐stained cryosections of the aortic root, aortic arch, and brachiocephalic artery. Reproduced with permission.^[^ ^35a^ ^]^ Copyright 2023, Royal Society of Chemistry.

Furthermore, different modification strategies have also been developed for MOF‐based drug delivery systems to overcome the limitations. For example, Liu et al. constructed a neutrophil membrane (NM)‐coated ZIF‐8 delivery platform for the antisense oligonucleotides (ASOs) (AM@ZIF@NM, **Figure** **8**a) targeting the ECs in atherosclerosis.^[^ ^35a^ ^]^ Utilizing the regulatory ability of miR‐155 for BCL6 in endothelial cells, delivery of anti‐miR55 can effectively reduce NF‐κB expression and eliminate inflammatory responses (Figure 8b).^[^ ^35a^ ^]^ Also, cellular membrane coating is a robust top‐down biomimetic approach that can help nanocarriers escape from the clearance of MPS. Meanwhile, MOFs not only possess a high loading capacity of cargo but also properly release ASOs into the target site after escaping from the endosome, therefore the nanoplatform produces significant mitigating and therapeutic effects on atherosclerosis (Figure 8c,d).^[^ ^35a^ ^]^

**Figure 9:**
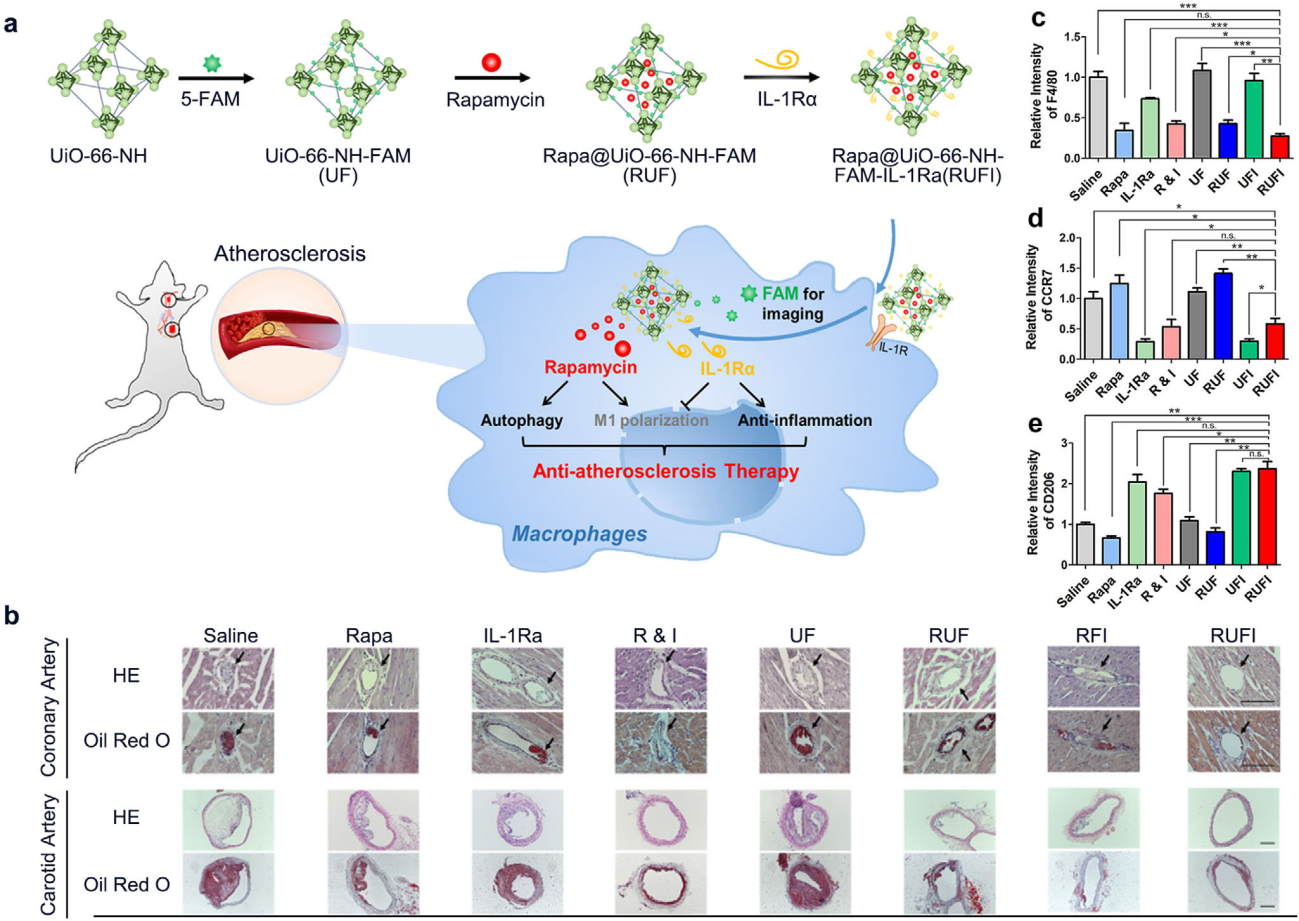
a) Preparation of the RUFI and the mechanism of the anti‐atherosclerosis process. b) H&E and Oil Red O staining of the atherosclerotic plaques in coronary arteries and carotid arteries. (Arrows indicate coronary arteries; Scale bar = 100 µm) c–e) Relative intensities of F4/80, CCR7, and CD206 compared to the saline group (^*^ *p* ≤ 0.05; ^**^ *p* ≤ 0.01; ^***^ *p* ≤ 0.001, n.s., not significant). Reproduced with permission.^[^ ^34a^ ^]^ Copyright 2023, Elsevier.

In addition, the membranes of red blood cells, other leukocyte cells, and platelets are also capable of coating nanocarriers like MOFs which provide a choice for the construction of more stable and effective drug delivery systems.^[^ ^82^ ^]^ On the basis of the cell membrane surface coating strategy, Liu and co‐workers developed a general coating strategy using phospholipids to synthesize a bilayer‐coated MOF NU‐901 recently.^[^ ^83^ ^]^ The MOF NU‐901 not only maintains good dispersion and long‐term stability in biological fluids, but also provides excellent encapsulation, targeted transport, and slow release of pemetrexed (a standard chemotherapeutic agent for mesothelioma).^[^ ^83^ ^]^ Another strategy to enhance the specificity of nanocarriers is conjugating the antagonist of inflammatory cytokine with the nanomaterials. Specifically, IL‐1RI, whose antagonist is IL‐1Rα, is an inflammatory cytokine that is highly expressed in inflammatory macrophages. According to the research of Xu and coworkers, UiO‐66‐NH MOFs modified by 5‐carboxyfluorescein (5‐FAM), rapamycin and IL‐1Rα (Rapa@UIO‐66‐NH‐FAM‐IL‐1Ra abbreviated as RUFI, **Figure** **9**a showed that the slow releasing of IL‐1Rα from the MOFs made these nanoparticles be endocytosed by inflammatory macrophages faster than MOFs without IL‐1Rα. Therefore, the interaction between IL‐1Rα on the MOFs and IL‐1RI on macrophages is effective in the targeting ability of the delivery flatform.^[^ ^34a^ ^]^ In vivo experiments have demonstrated the efficacy of RUFI in inhibiting atherosclerosis by reducing M1 macrophages and promoting M2 macrophages (Figure 9b–e).^[^ ^34a^ ^]^ Furthermore, these experiments have shown that RUFI combines the advantages of rapamycin and IL‐1Rα to reduce total macrophages in plaques and regulate the polarization of plaque macrophages, thereby achieving an excellent therapeutic effect on atherosclerosis.^[^ ^34a^ ^]^

### 3.2 Therapeutic Gas Delivery

**Figure 10:**
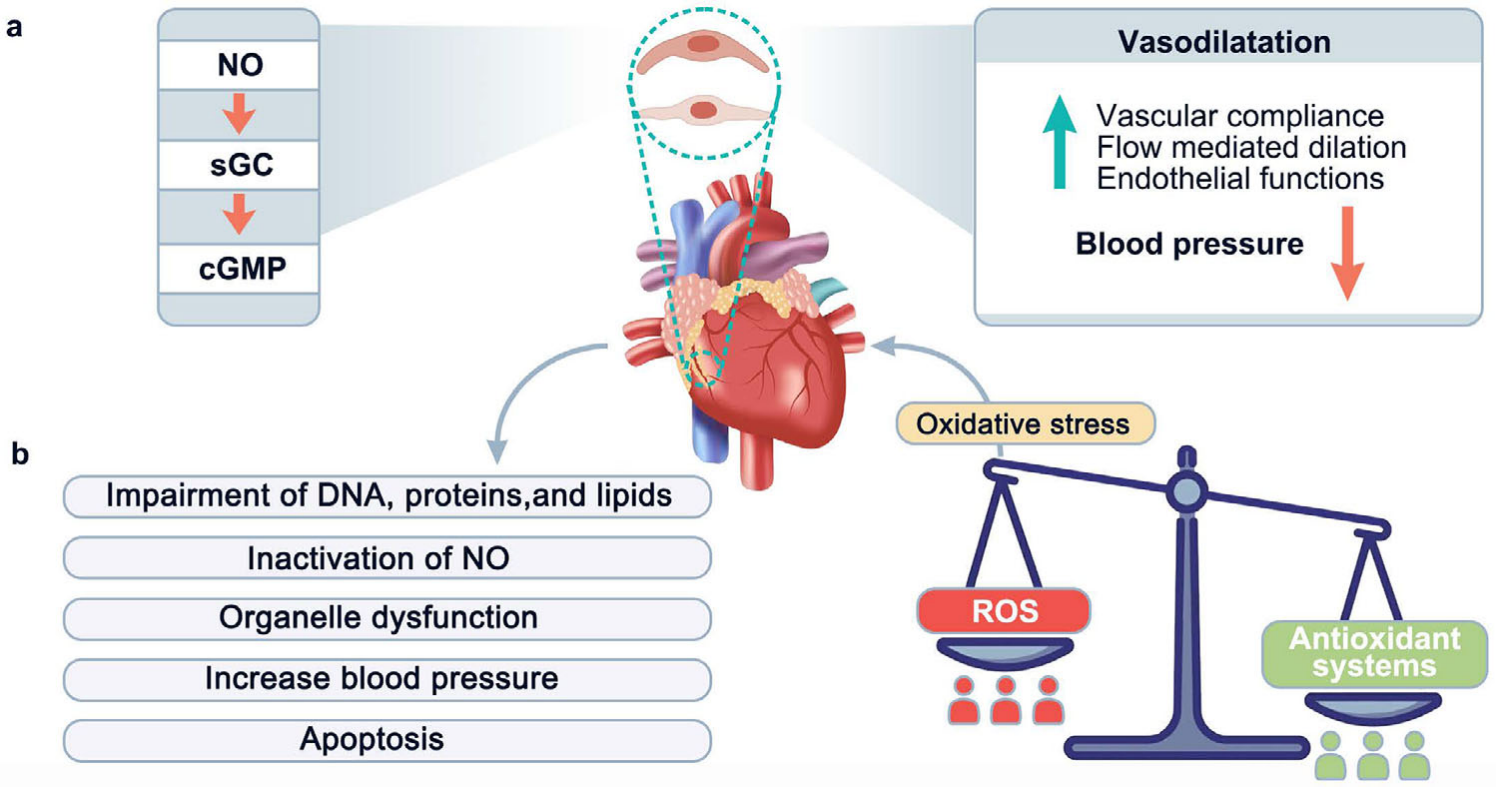
a) The vasodilatory effect of NO on the cardiovascular system. b) The over‐production of ROS causing excessive oxidative stress and histopathological damage to the heart.

Therapeutic nitric oxide (NO) gas, as a signaling molecule in the cardiovascular system, plays an important role in cardiovascular homeostasis. In particular, it can contribute to endothelial or smooth muscle cell‐mediated vasodilation and increase blood flow by activating soluble guanylate cyclase (sGC) and increasing intracellular levels of cyclic guanosine monophosphate (cGMP) which is shown in **Figure** **10**a.^[^ ^84^ ^]^ Although NO is a simple gas molecule, the therapeutic actions of it are complicated. For example, the roles and functions of NO highly depend on the concentrations, which means higher or lower concentrations may all induce deleterious effects like the development of CVD.^[^ ^85^ ^]^ In addition, the limited biological half‐life of NO is a severe obstacle to the efficacy of treatment.^[^ ^84a^ ^]^ Meanwhile, the reaction between superoxide radicals (O_2_^•−^) and NO can induce DNA damage and lipid peroxidation.^[^ ^86^ ^]^ Therefore, the effectiveness of therapeutic NO delivery is not only related to the amount loaded or delivered of NO by carriers. In other words, the protective delivery process and the right amount of proper NO released in the target site are the keys to the delivery of therapeutic gas at present. In the research of delivery for therapeutic gas, many materials have been used as carriers to load NO gas, such as MOFs, inorganic porous materials, polymer nanoparticles, silicon nanoparticles, dendrimers, micelles, liposomes, and others.^[^ ^87^ ^]^ Among them, MOFs are burgeoning materials with porous structures, high surface area, and open metal sites, which can adsorb NO chemically on their Lewis acid sites and extend the half‐life of NO.^[^ ^88^ ^]^

**Figure 11:**
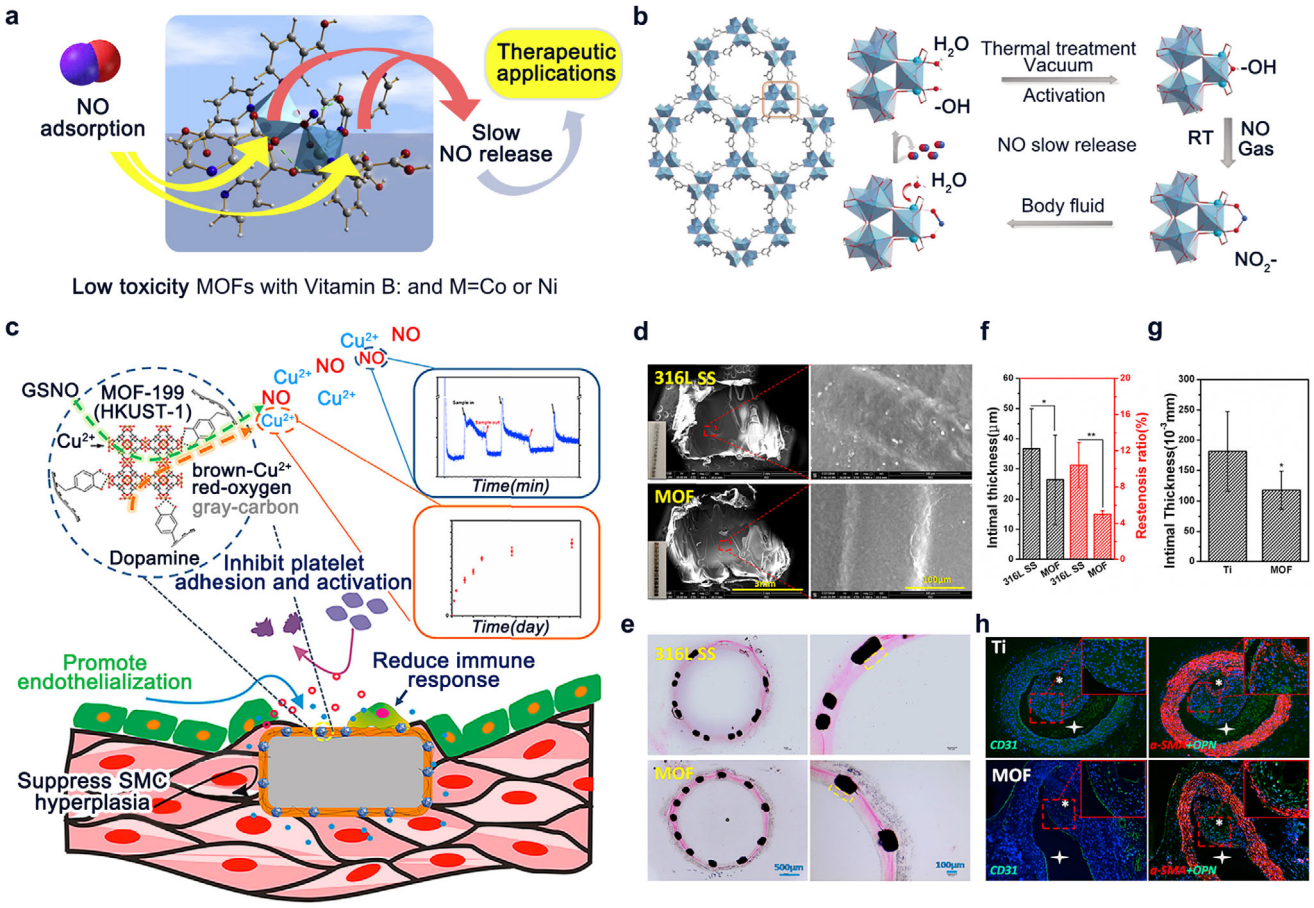
a) The slow release of NO by NO‐loaded MOFs with vitamin B_3_. Reproduced with permission.^[^ ^91^ ^]^ Copyright 2017, Elsevier. b) MIP‐177 structure and NO binding/release mechanism. Reproduced with permission.^[^ ^93^ ^]^ Copyright 2020, Wiley‐VCH. c) Cu‐MOFs‐based surface coating of cardiovascular stents for adaptable, steady NO release and desirable copper ion delivery which has shown synergy effect on promoting endothelialization, inhibiting platelet adhesion and activation, suppressing intimal hyperplasia, and reducing immune response. d–h) Rabbits (d–f) and SD rats (g,h) implantation experiments in vivo: d) SEM of bare and nano Cu‐MOF‐immobilized 316 L SS stents after a four‐week implantation. e) Effect of the bare and nano Cu‐MOFs‐immobilized 316 L SS stents on ISR assessed by histomorphometry analysis. f) Statistical analysis of the neointimal thickness and restenosis rate. g) Intimal thickness of Ti and nano Cu‐MOFs‐immobilized Ti wire (d  =  0.1 mm) after implanting into the abdominal aorta of rats for four weeks. h) Immunofluorescence staining of the abdominal aorta after implantation for four weeks. Data are presented as the means ± SD (*n* ≥ 4) and analyzed using one–way ANOVA, ^*^ *p* < 0.05, ^**^ *p* < 0.01, and ^***^ *p* < 0.001. Reproduced with permission.^[^ ^30a^ ^]^ Copyright 2019, Elsevier.

Early on, it was found that some MOFs have great gas adsorption capabilities including NO gas. Morris's group used a copper benzene tricarboxylate MOF material (HKUST‐1) that can bind NO through the empty copper metal sites in HKUST‐1.^[^ ^88a^ ^]^ Later, they established two different porous MOFs containing metal Co and Ni (Co‐MOF and Ni‐MOF) with 2,5‐dihydroxyterephthalic acid as the organic linker, respectively.^[^ ^88b^ ^]^ Although both MOFs exhibited high gas adsorption capacity, storage stability, long shelf life, and triggered release under biofluids, the biotoxicity associated with the metals cannot be ignored, especially for Co. Since then, a range of MOFs with better biocompatibility have been developed for NO adsorption with controlled release. A microporous MOF (BioMIL‐3) constructed by the bioactive calcium and 3,30,5,50‐azobenzenetetracarboxylate ligand, has been synthesized as a fascinating candidate for NO gas controlled release in the biomedical field.^[^ ^89^ ^]^ Theoretically, BioMIL‐3 contains coordinatively unsaturated Lewis acid sites which are capable of absorbing and releasing NO gas at the biological level with relatively low toxicity, but the total amount of NO actually adsorbed was lower than the theoretical amount of unsaturated Ca^2+^ metal sites, indicating that the efficiency of NO entry into the pores was relatively low. This inefficiency may be caused by the narrow pore sizes in the flexible structure of the frameworks.^[^ ^89^, ^90^ ^]^ Synthesized MOFs with non‐toxic ligands is another way to enhance the biocompatibility of MOFs. For instance, vitamin B_3_ (niacin or nicotinic acid) as a more biocompatible ligand can connect to different metals like Ni and Co to form a porous structure.^[^ ^91^ ^]^ And the results have confirmed that the toxicity of these MOFs is really low when the concentrations are below 180 µg cm^−3^, and these MOFs follow a slow‐release pattern of biologically active NO in either the gas or liquid phase (**Figure** **11**a).^[^ ^91^ ^]^

At present, most of the research deals with a wet gas release for anti‐thrombogenic or antibacterial applications,^[^ ^92^ ^]^ while studies in a liquid phase (buffers) especially in biological fluids are scarce. Pinto et al. established a titanium‐based MOF (MIP‐177, MIP stands for the materials of the Institute of Porous Materials of Paris) whose chemical formula is Ti_12_O_15_(mdip)_3_(OH)_6_(H_2_O)_6_ (mdip = 3,3′,5,5′‐tetracarboxydiphenylmethane) with high NO storage capacity, excellent biocompatibility and long‐term NO release, and the microporosity and NO binding/release mechanism of MIP‐177 are shown in Figure 11b.^[^ ^93^ ^]^ NO release times of most MOFs are in excess of several hours or even 40 h under humid nitrogen or under vacuum,^[^ ^94^ ^]^ but the times are greatly reduced in liquid environments, especially biological media due to the excessive rapid release of NO and fast degradation of metal carboxylate MOFs in biological fluids. The researchers suggest that the slow release of NO from MIP‐ 177 have possibly something to do with its chelation of nitrite inside the framework structure since nitrite can be decomposed slowly by reacting with water and participating in the rehydration process of metal sites, resulting in a stable release of NO in the liquid phase.^[^ ^93^ ^]^ Moreover, Fe‐based MOFs have also been explored for NO delivery in biological media for biomedical applications. Recently, Pinto et al. developed a new stable Fe bisphosphonate MOF (MIP‐210(Fe)) containing p‐xylenediphosphonic acid and Fe^3+^ which can achieve long‐term NO release over 72 h.^[^ ^95^ ^]^ Fe^3+^ metal site preferentially coordinates to NO over water and usually exhibits good biocompatibility in biological media,^[^ ^96^ ^]^ also Phosphonate MOFs have better stability than other carboxylate MOFs due to the stronger bonds between phosphonates and metal atoms.^[^ ^97^ ^]^ Therefore, MOFs formed by Fe^3+^ and phosphonate can theoretically combine the advantages of both which have great prospects for biomedical applications. In two different cell lines, low concentrations of NO‐loaded MIP‐210(Fe) resulted in higher cell viability than cells with pure MIP‐210(Fe), demonstrating that NO released from MOFs has a positive effect on cell growth.^[^ ^95^ ^]^ Although the above studies are oriented toward the biomedical field, they are still limited at a cellular level to exert an angiogenic effect, we would prefer that these MOFs can be practically applied in the field of CVD in the future.

The emerging way for the application of MOF‐based NO gas delivery in CVD treatment is MOF‐coated cardiovascular stents.^[^ ^30^ ^]^ Although cardiac stent surgery is the most effective treatment for atherosclerosis, the biosafety of the stents in the long term is still a matter of concern due to in‐stent restenosis (ISR) and late stent thrombosis (LST).^[^ ^30^, ^98^ ^]^ Whereas in normal ECs, sustained production of NO is effective in combating some problems such as inflammation and platelet activation. Attempts have been made to mimic the cellular secretion of NO to enable cardiovascular stents to sustainably release NO to counteract the problems associated with ISR and LST, thereby improving the long‐term safety of cardiovascular stents.^[^ ^14^, ^32^ ^]^ Some copper‐based MOFs (Cu‐MOFs) can catalyze the decomposition of endogenous NO donors such as S‐nitrosothiols (RSNOs)^[^ ^99^ ^]^ and S‐nitrosoglutathoine (GSNO)^[^ ^14^ ^]^ to produce NO. Thus, Cu‐MOFs have been wrapped around the surface of medical materials to create a novel biocomposite that catalytically produces NO in the presence of endogenous NO donor, which further reduces the side effects of cardiovascular stents.^[^ ^30a^ ^]^ Besides, some studies confirmed that copper ions stimulate the growth and function of ECs through upregulating the expression of vascular endothelial growth factor.^[^ ^100^ ^]^ However, the surface of Cu‐MOFs needs to be treated to activate the metal surface to generate sufficient ionic coordination centers, which may be an important reason for limiting the development of Cu‐MOFs in cardiovascular stents.^[^ ^30a^ ^]^ To address these issues, Fan et al. immobilized Cu‐MOFs on the surface of cardiovascular scaffolds using a polydopamine layer with strong adhesion, which significantly enhanced intravascular anti‐inflammation, anticoagulant, and anti‐hyperplasia effects under the synergistic effect of Cu ions and NO release (Figure 11c).^[^ ^30a^ ^]^ The findings demonstrated that stents coated with nano Cu‐MOFs exhibited the capacity to adhere to a substantial number of erythrocytes and platelets, thereby effectively mitigating the risk of delayed endothelialization and thrombosis (Figure 11d). Additionally, the thickness of the neointima and the restenosis rate was notably reduced (Figure 11e,f). Furthermore, the experimental group treated with Cu‐MOFs exhibited an intact endothelial monolayer and a thinner neoendothelium compared to the bare Ti wire group, suggesting enhanced reendothelialization and reduced ISR. The in situ catalyzed NO generation and copper ion delivery by nano‐Cu‐MOFs‐immobilized coatings on cardiovascular stents demonstrated a significant capacity to promote reendothelialization and mitigate ISR.^[^ ^30a^ ^]^

Meanwhile, Zhao et al. constructed a copper‐based surface‐attached MOFs (Cu‐SURMOFs) by the method of layer‐by‐layer self‐assembly with Cu(II) benzene‐1,3,5‐tricarboxylate (CuBTC) whose NO flux was stable even if the amount of NO donor was reduced, and the cardiovascular stents coated with Cu‐SURMOFs showed great implantation result after 4 weeks.^[^ ^14^ ^]^ Of course, MOFs can be used as coatings, but can also be directly inserted into biomaterials during the shaping process. For example, Cu‐MOFs were embedded into polycaprolactone (PCL) fibers using an electrospinning technique, and the cardiovascular stents based on this new type of PCL exhibited a better implantation effect so that implanted materials remained active for a long time.^[^ ^32^ ^]^ Overall, as an effective protective layer of cardiovascular stents, Cu‐MOFs had greatly reduced the side effects brought by stent implantation and enhanced the recovering ability of blood vessels. The experimental results indicated that Cu‐MOFs could be used as an effective class of biomaterial coatings, which was of great significance for the development of cardiovascular stents.^[^ ^30^, ^32^ ^]^

### 3.3 Nanozyme

**Figure 12:**
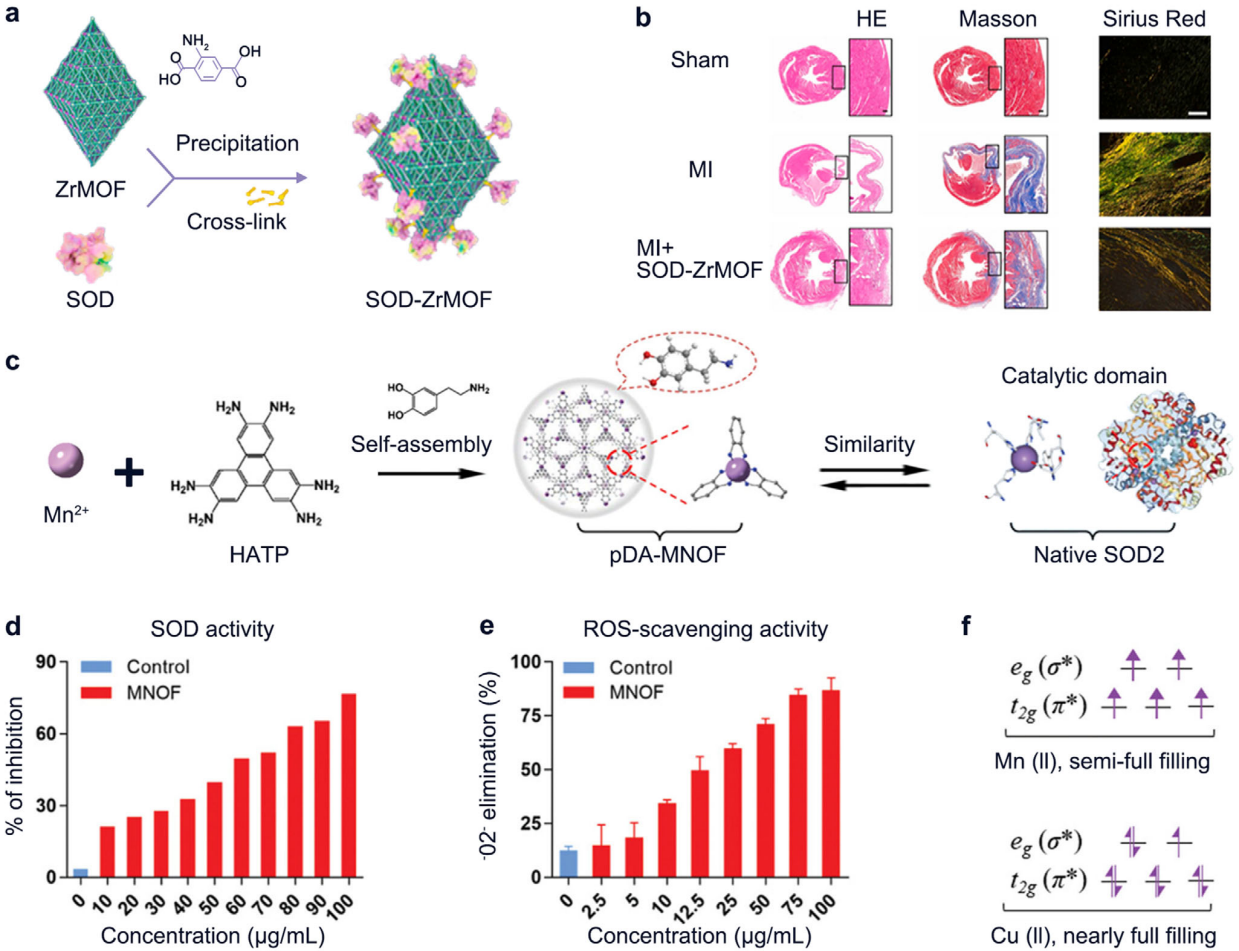
a,b) Schematic illustration of the construction of SOD‐ZrMOF via physical absorption approach; The efficacy of SOD‐ZrMOF in protecting cardiac function by decreasing infarct area and restraining pathological myocardial remodeling 28 days after MI: HE, Masson trichrome staining (Scale bar: 200 µm) and sirius red staining (Scale bar: 100 µm) were used to observe the degree of MI and fibrosis (*n* = 6). Reproduced with permission.^[^ ^13^ ^]^ Copyright 2022, Elsevier. c) Schematics of fabrication of pDA‐MNOF that possessed a bioinspired structure mimicking the catalytic domain of native SOD2. d–f) SOD‐like catalytic activity of MNOF; ROS scavenging effect of MNOF; 3D electron occupancy of t_2_ _g_ (π^*^) and eg (σ^*^) antibonding orbitals associated with transition metals for Mn (II) and Cu (II). Reproduced with permission.^[^ ^112^ ^]^ Copyright 2023, Wiley‐VCH.

Oxidative stress is a key factor involved in the development of CVDs.^[^ ^101^ ^]^ As an essential part of oxidative stress, the series of by‐products in natural cell metabolism with chemical oxidative activity called reactive oxygen species (ROS) play important roles in the regulation of the physiological process of aerobic life. Usually, the ROS are mainly composed of various free radicals such as hydroxyl radical (OH^•−^) and superoxide anion (O_2_^•−^) and nonradicals such as hydrogen peroxide (H_2_O_2_), singlet oxygen (^1^O_2_), peroxynitrite (ONOO^−^) and hypochlorous acid (HOCl).^[^ ^102^ ^]^ Low levels of ROS are significant for cell development and signaling,^[^ ^103^ ^]^ but over‐production of ROS from cellular metabolism may cause excessive oxidative stress and histopathological damage which leads to severe health problems including CVDs (Figure 10b).^[^ ^104^ ^]^ Therefore, regulating and scavenging ROS levels in vivo is a feasible way to alleviate and treat related diseases. The most intuitive design pathway for MOFs‐based nanomedicines is to utilize the ability of antioxidant enzymes to scavenge ROS by immobilizing the antioxidant enzymes on the surface or inside of the MOFs, and the enzymes are delivered to the site of inflammation for a further therapeutic and alleviating the inflammatory response in CVDs. In acute myocardial infarction, the increase of reactive oxygen species (ROS) is a key factor leading to excessive cardiac injury after the onset of the disease. Therefore, researchers addressed this issue by coupling superoxide dismutase (SOD) to porous nanomaterial Zr MOF using a crosslinking method (**Figure** **12**a).^[^ ^13^ ^]^ The obtained SOD‐ZrMOF nanocomposites demonstrate good biocompatibility, which could protect mitochondrial function, reduce cell death, and weaken inflammation by effectively clearing ROS and inhibiting oxidative stress. Meanwhile, SOD‐ZrMOF can effectively reduce infarct area, protect heart function, promote angiogenesis, and inhibit pathological myocardial remodeling (Figure 12b). Therefore, these nanocomposites have great potential in the treatment of acute myocardial infarction.^[^ ^13^ ^]^

However, natural enzyme suffers from an unstable protein structure, which causes them to be more prone to breakdown or denaturation during the process of both preservation and delivery, rendering the catalytic reaction impossible.^[^ ^105^ ^]^ In contrast, synthetic enzymes have been explored to replace the role of natural enzymes, which can achieve the same catalytic effect and therapeutic purpose while ensuring the stability of the catalysts.^[^ ^106^ ^]^ MOF‐based nanozyme is an artificial enzyme that possesses the catalytic effect by mimicking the active structural domains of enzymes. For example, Fe‐based MOF is a popular MOF‐based nanozyme that can mimic the catalytic activity of peroxidase, of which NH_2_‐MIL‐88B(Fe) is a typical representative. The structure of NH_2_‐MIL‐88B(Fe) is built up from trimers of Fe(III) octahedra that are further linked by 2‐aminoterephthalic acid ligands.^[^ ^107^ ^]^ And the iron atom in each octahedron acts as a catalytic center making NH_2_‐MIL‐88B(Fe) possess remarkable peroxidase‐like activity in acidic environments.^[^ ^107^, ^108^ ^]^ Besides, compared to natural enzymes, the nanoscale size of nanozyme can enhance its deliverability in organisms, and exhibit a more flexible and broader substrate spectrum.^[^ ^106^, ^109^ ^]^ ROS‐scavenging nanozymes can protect cells from ROS damage, and have been proven to be a powerful tool for CVD treatment.^[^ ^110^ ^]^ Manganese‐based MOFs (Mn‐MOFs) can exhibit SOD‐like catalytic activity because SOD is a series of metal‐dependent enzymes including manganese‐dependent SOD (SOD2) and copper/zinc‐dependent SOD (SOD1).^[^ ^111^ ^]^ As shown in Figure 12c, a dopamine‐modified manganese‐organic framework (pDA‐MNOF) was utilized to upregulate the activities of two endogenous antioxidant enzymes in vivo.^[^ ^112^ ^]^ The results showed that MNOF had a dose‐dependent SOD‐like activity, i.e., the ROS scavenging activity was concentration‐dependent (Figure 12d,e). In particular, the choice of metal nodes in the MOF structure is also particularly important for the ROS scavenging effect. Electron orbital analysis also showed that the d‐electron orbitals of Mn (II) were half‐filled compared to those of Cu (II), which were almost completely filled, and thus the nano‐enzymatic activity of Cu‐MNOF would be affected and decreased (Figure 12f).^[^ ^112^ ^]^

**Figure 13:**
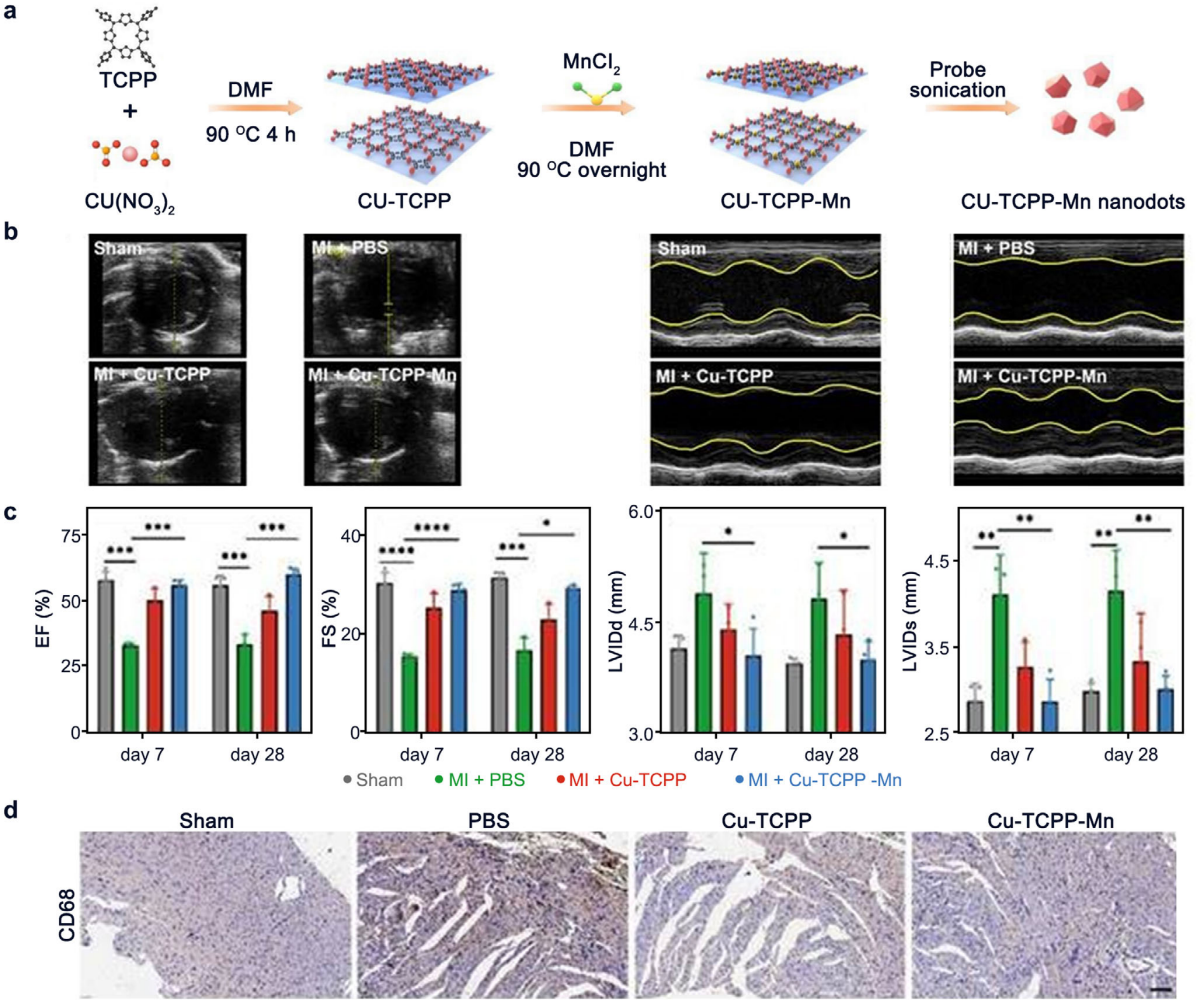
a) Illustration of Cu‐TCPP‐Mn nanozyme. b) Representative echocardiographic images and corresponding M‐mode images of MI mouse models on day 7 after treatments. c) Ejection fraction (EF) and fractional shortening (FS) results of heart obtained by echocardiography after different treatments at day 7 and day 28, respectively (*n* = 3–4 per group); Left ventricular end diastolic dimension (LVIDd) and left ventricular end systolic dimension (LVIDs) obtained by echocardiography after different treatments at day 7 and day 28, respectively (*n* = 3–4 per group). d) Heart tissue sections stained with CD68 (brown) and DAPI (blue) in MI mice treated with PBS, Cu‐TCPP, Cu‐TCPP‐Mn. Scale bar, 100 µm. Reproduced with permission.^[^ ^38^ ^]^ Copyright 2023, Ivyspring International.

Besides, the abundance of metal ions in MOFs not only mimics the catalytic activity of the enzyme but also the intrinsic interactions between metal ions can enhance the overall activity. For example, Wu et al. constructed a SOD‐like MOF‐818 nanozyme containing both Cu and Zr and found that the presence of Zr modulated the active site in the catalytic process, lowered the energy barrier, and enhanced the cyclic conversion activity between Cu(II) and Cu(I), thus enhancing the catalytic activity of natural SOD1.^[^ ^37^ ^]^ Another bimetallic MOF‐based nanozyme contains Cu and Mn (Cu‐TCPP‐Mn) have also been studied for myocardial repair and remodeling.^[^ ^38^ ^]^ Cu‐TCPP‐Mn (**Figure** **13**a) has shown great performance in both SOD‐ and CAT‐like activities which provide synergistic ROS scavenging effect inhibiting inflammation, reducing myocardium fibrosis, and enhancing constructive remodeling and vascularization, and preventing myocardium from damage.^[^ ^38^ ^]^ It was shown that Cu‐TCPP‐Mn treatment for 7 days effectively alleviated the thinning of the anterior and posterior walls of the heart as well as the systolic and diastolic cardiac dysfunction caused by myocardial infarction surgery (Figure 13b), while ejection fraction (EF), fractional shortening (FS), diastolic left ventricular internal diameters (LVIDd) and systolic left ventricular internal diameters (LVIDs) were all significantly improved (Figure 13c).^[^ ^38^ ^]^ In addition, the infiltration of neutrophils (CD45^+^) and macrophages (CD68^+^) was significantly reduced in the Cu‐TCPP‐Mn group (Figure 13d).^[^ ^38^ ^]^ Therefore, Cu‐TCPP‐Mn nanozymes have anti‐ROS and antioxidant functions that can improve cardiac function after myocardial infarction and promote cardiac repair, thus playing a protective and therapeutic role in the myocardium.

Recently, a MOF‐based integrated cascade nanozyme Pt@PCN222‐Mn mimicking the cascade catalytic reaction of mitochondria and peroxisome has been developed to eliminate excess ROS. This nanozyme mimics SOD activity by combing metal Zr with manganese porphyrin [5,10,15,20­tetrakis(4­carboxyphenyl)­porphyrinato]‐Mn(III) chloride (TCPP‐Mn), an organic ligand capable of converting oxygen radicals to H_2_O_2_, followed by inserting platinum (Pt) nanoparticles to mimic CAT activity. The results demonstrated that the metal‐free porphyrin TCPP did not exhibit SOD‐like activity, whereas both TCPP‐Mn and PCN222‐Mn exhibited strong SOD‐like activity, suggesting that Mn plays a key role in mimicking SOD‐like activity in porphyrins. In vivo experiments showed that the cascade nanozyme exhibited excellent anti‐inflammatory and antioxidant therapeutic effects in mice compared to mice treated with an unintegrated mixture of the two nanozymes.^[^ ^113^ ^]^

Except for metal Mn, many other metal elements can also be applied in the synthesis of anti‐inflammatory nanozyme.^[^ ^28^ ^]^ For instance, MIL‐47 (V), a microporous vanadium(IV) terephthalate, was reported in 2002.^[^ ^114^ ^]^ This MOF was shown to mimic the activity of glutathione peroxidase (GPx), which is also a type of antioxidant enzyme, for the therapeutic application was first reported in 2020.^[^ ^28b^ ^]^ The principle of GPx limiting the deleterious effects of ROS is the conversion of H_2_O_2_ to H_2_O. However, the natural GPx usually suffers from low stability and poor availability, which have hampered its biomedical applications. Therefore, inspired by protein engineering, Wu et al. mimicked GPx activity by altering the substituent X (X = F, Br, NH_2_, CH_3_, OH, and H) in the ligand of MIL‐47(V) to form the new MIL‐47(V)‐X (Figure 6e). MIL‐47(V)‐NH_2_ showed the best antioxidant effect in vitro as a ROS scavenger which protected cells from oxidative damage, and anti‐inflammatory experiments demonstrated its broad‐spectrum anti‐inflammatory effects.^[^ ^28b^ ^]^ The study of MOF‐based nanozymes strongly expands the scope of biomimetic MOFs, making this class of antioxidant nanozymes an extension of current research in the nanomedicine field.

### 3.4 Other Strategies

Besides, the treatment of ischemic conditions associated with CVD is particularly important. Proangiogenesis plays a key role in the treatment of ischemic heart disease, but it is difficult to meet the safety, cost, and other elements of evaluation of related drugs. Metal ion‐based therapies have emerged as an effective therapeutic strategy due to the important role of metal ions in regulating adenosine triphosphate (ATP) synthesis, enzyme activation, signaling pathways, and cellular behavior.^[^ ^115^ ^]^ However, such treatment relies on high dose intake to achieve effective therapeutic effects, and the use of metal ions carries strong toxicological risks.^[^ ^116^ ^]^ Thus, the introduction of little amount of metal ions with angiogenesis‐promoting activity into the structure of MOFs has the potential to extend the development of drugs promoting angiogenesis due to the dual advantages of metal ions and MOFs. Gao et al. designed a novel proangiogenic drug by synthesizing a Zn‐MOF called ZIF‐90 with Zn^2+^ as a metal node. They decorated this MOF with EC‐targeted and mitochondria‐localizing‐sequence peptides which further enhanced the specificity of the Zn‐MOFs. Their results confirmed that these Zn‐MOFs indirectly stimulate cardiomyocyte morphology and angiogenesis through the PI3K/Akt/eNOS pathway without the need for other therapies, which successfully combine the advantages of low dosage, minimum toxicity, low cost, high efficiency, and scalable mass production.^[^ ^36^ ^]^ Therefore, combining biologically active metal ions with MOFs is expected to be applied to the treatment of ischemic diseases.

## 4 Biocompatibility of MOFs

Although hundreds of studies have been conducted to investigate the medical applications of MOFs in CVD or other related conditions, there are questions about the biocompatibility or toxicity of MOFs in vivo that have not been fully validated especially in the long term. The variety of MOFs contributes to the fact that they exhibit different biocompatibility in different environments no matter in vitro or in vivo. Wuttke et al. evaluated six different kinds of MOFs within the series of MIL and Zr‐based MOFs. Then, they applied them to respective applications such as systemic drug delivery, local lung‐specific drug delivery, and coating of medical materials.^[^ ^117^ ^]^ They found that the nano‐safety of MOFs can be diametrically opposed in different applications related to an intrinsic discrepancy of the cell types and the presence or absence of MOF surface modifications. For example, MIL‐100(Fe) has shown good biocompatibility in primary human endothelial cells for systemic drug delivery and primary human gingiva fibroblasts for coating of dental implants, but in lung epithelial cells, lung immune cells and neural cells MIL‐100(Fe) had produced significant adverse effects like pronounced activation of inflammations and stronger inhibitory effect on metabolic activity of cells.^[^ ^117^ ^]^ Meanwhile, MIL‐101(Cr) coated with lipids such as 1,2‐dioleoyl‐sn‐glycero‐3‐phosphocholine (DOPC) showed better biocompatibility in lungs than naked MIL‐101(Cr).^[^ ^117^ ^]^ Thus, the selection of MOFs with high biocompatibility and biosafety in specific environments is particularly important for medical applications, and the toxicity of MOFs in the cardiovascular system should be thoroughly investigated.

In addition, the role of metal elements in MOFs is also noteworthy in biocompatibility and toxicity.^[^ ^118^ ^]^ For instance, it has been shown that when serum zinc levels are greater than 100 µg dL^−1^, the incidence of CVD would increase significantly with the increasing serum zinc levels.^[^ ^119^ ^]^ These results have greatly increased the skepticism about Zn‐based MOF materials for medical applications, such as ZIF‐8,^[^ ^69a,b^ ^]^ which degrade fast under acidic conditions and release a large amount of zinc ions (and 2‐methylimidazole). Kota et al. investigated the effects of low‐dose ZIF‐8 nanoparticles on vascular smooth muscle cells (VSMC) morphology, actin organization, and contractility.^[^ ^120^ ^]^ Although the concentration of zinc ions was kept within the range considered “safe”, the cells were not unaffected, e.g., the actin cytoskeleton and cellular morphology have changed, as the presence of zinc ions disrupted the actin assembly process, which further affected the phenotype of the cells.^[^ ^120^ ^]^ Similar results have also been found in human aortic endothelial cells (HAECs) when “non‐cytotoxic” doses of ZIF‐8 nanoparticles were delivered into the cells.^[^ ^121^ ^]^ Therefore, intravenous injection of low doses of MOFs nanoparticles containing zinc ions like ZIF‐8 may still pose a cardiovascular safety risk, while a controlled low dosage of MOFs with zinc ions can still contribute to the mitigation and treatment of the ischemic milieu in CVD.^[^ ^36^ ^]^

**Figure 14:**
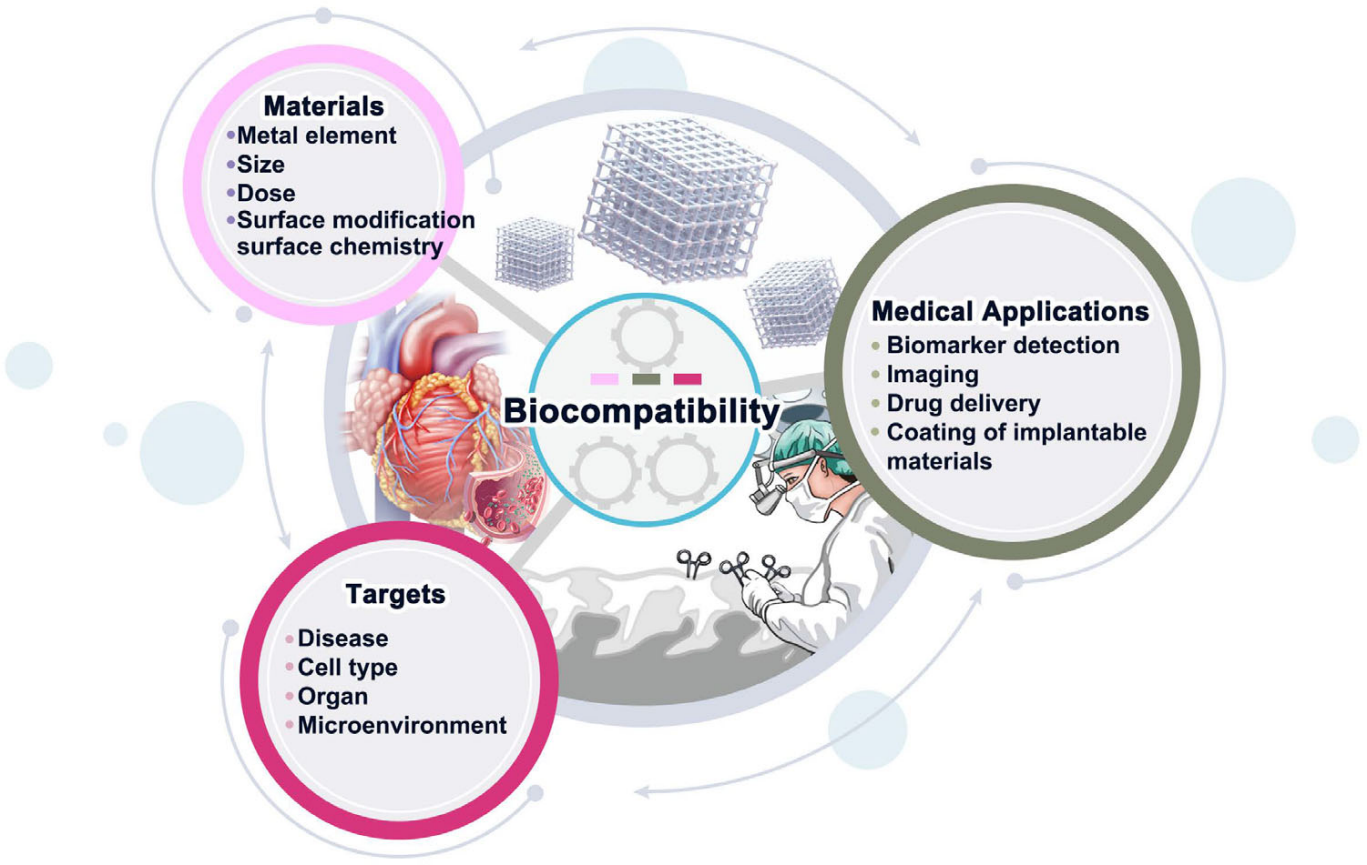
Multiple factors combine to influence the biocompatibility of MOF as a material for disease diagnosis and treatment.

Also, the rate at which MOFs are cleared from the bloodstream greatly influences its toxicity. The rate of clearance from the blood is mainly related to the hydrophobic‐hydrophilic balance of ligands because MOFs with hydrophobic ligands can only be cleared slowly by the liver and spleen and cannot be excreted directly through the urine as quickly as MOFs with hydrophilic ligands.^[^ ^122^ ^]^ In addition to this, factors such as dose,^[^ ^120^, ^121^ ^]^ size, morphology,^[^ ^122^ ^]^ surface chemistry,^[^ ^123^ ^]^ and stability^[^ ^124^ ^]^ all may affect the biocompatibility or toxicity of MOFs.^[^ ^125^ ^]^ At the same time, in spite of the nature of the material itself, other factors like different therapeutic and diagnostic purposes and disease characteristics will all collectively affect the biocompatibility of the applications of MOFs in the diagnosis and treatment of disease (Figure 14). However, most of these studies did not investigate in the cells or systems related to CVD, so the toxicity studies specifically focusing on MOFs in the cardiovascular environment are insufficient, and more research should be focused on this in the future to further advance the applications of MOFs in the diagnosis and treatment of CVD.

## 5 Conclusion and Outlook

With their rapid development, MOFs have played an important role in the field of nanomaterials for biomedical applications. Especially in the treatment and diagnosis of CVD. Due to their flexibility, high porosity, high specific surface area, controllable structure, and diverse functions, MOFs can be used as carriers to protect diagnostic or therapeutical molecules and deliver them specifically to the target sites. In addition, some functional metal ions or organic molecules can be incorporated into the synthesis of MOFs as metal nodes or organic ligands, respectively, which makes MOFs obtain biological function for diagnostic and therapeutic purposes. The cardiovascular system is the most important component of blood transportation in the body, and the complex distribution of the vasculature, diverse blood flow, and biological environment pose a significant challenge to the diagnosis and treatment of related diseases. MOFs are now emerging in CVD theranostics, bringing new hope for biomedical applications due to their versatile types, forms, sizes, and properties.

The unique value of MOFs in medicine is mainly supported by their structural diversity, including the larger specific surface area and tunable pore size, which are derived from the variety of metal ions and organic ligands.^[^ ^126^ ^]^ Typically, the specific surface area of MOFs can reach more than 1000–10 000 m^2^ g^−1^,^[^ ^127^ ^]^ which is much higher than that of other porous materials, such as hydrogels, liposomes, and micelles. Therefore, MOFs are suitable for high‐dose drug delivery or simultaneous delivery of various drugs, allowing for synergistic therapy.^[^ ^128^ ^]^ Certainly, compared with porous materials, MOFs have many limitations, including biocompatibility, toxicity of degradation products, and preparation cost,^[^ ^66^ ^]^ and these factors have been the prerequisites bothering the extensive application of MOFs in the medical field. Therefore, it is particularly important to select suitable MOFs for targeted delivery with non‐toxicity or low toxicity. Moreover, high drug loads are two‐sided for the treatment of disease. In terms of advantages, high loading reduces the concentration of the MOFs used, which in turn reduces potential toxic effects and biocompatibility risks,^[^ ^129^ ^]^ as well as lowering the cost of preparation of the MOFs.^[^ ^130^ ^]^ Meanwhile, the therapeutic effect is enhanced by delivering more drugs. However, this high drug loading capacity can also present a number of challenges, including the potential for explosive or delayed drug release,^[^ ^131^ ^]^ impaired structural stability of the MOFs. Therefore, drug loading capacity must be based on the actual application and assessed against a range of factors. In addition, from a clinical translational perspective, there are still many challenges to the application of MOFs to the diagnosis and treatment of cardiovascular diseases. For example, 1) stability in physiological environment. The instability of MOFs has been a major drawback limiting their application. Due to the complexity of environmental factors in blood tissues, it is easy for MOFs loaded with drugs to experience uncontrolled release, which will lead to the inability to achieve precise delivery. Therefore, it is necessary to combine MOFs that are easily degradable with some biological molecules to ensure their stability and biocompatibility. 2) Scalability and reproducible manufacturing. Since the MOFs synthesized in the early stage were prepared in small systems, in the later stage of the transformation process, MOFs need to be prepared in large quantities. It is necessary to ensure that the morphology and properties of the large quantities of the products are consistent with those of the small systems, so as to realize reproducible large‐scale preparation, and to lay the foundation for the later stage of the clinical application.

Certainly, MOFs also provide some new perspectives for the treatment and diagnosis of CVD. For example, therapeutic gas (NO) delivery, treatment integration (simultaneous loading of contrast agent and drug), and precision‐controlled release (responsive release). At present, the types of MOFs used for the diagnosis and treatment of CVD are still limited, because CVD is complicated and has different etiologies, and therapeutic targeting is not as consistent as it is in the case of cancer. Therefore, while developing new medical materials, we should also pay attention to the etiology and characteristics of the disease, so as to promote the development of MOF in the diagnosis and treatment of CVD by “adapting to the local conditions.” Furthermore, the use of cutting‐edge technology like intelligent machine learning can be used to screen out the type and structure of the MOFs efficiently and analyze the mechanism of their action in depth, which can further promote the design and construction of MOFs as biomedical nanomaterials.

## Conflict of Interest

The authors declare no conflict of interest.
